# Gut microbiota metabolic reprogramming drives the development of metabolic diseases in the host

**DOI:** 10.1080/19490976.2026.2644681

**Published:** 2026-03-14

**Authors:** Yanrong Wang, Beibei Huang, Xue Wei, Yuanyuan Guan, Lingru Li, Yanfei Zheng, Wenlong Sun

**Affiliations:** a School of Life Sciences and Medicine, Shandong University of Technology, Zibo, Shandong, People's Republic of China; b National Institute of Traditional Chinese Medicine Constitution and Preventive Medicine, Beijing University of Chinese Medicine, Beijing, People's Republic of China

**Keywords:** Gut microbiota, metabolites, metabolic reprogramming, metabolic diseases

## Abstract

Metabolic diseases pose a major global health challenge, the pathogenesis of which centers on “metabolic reprogramming”; that is, the adaptive or pathological rewiring of metabolic pathways. Emerging evidence indicates that gut microbiota dysbiosis triggers its metabolic reprogramming prior to host disease onset and plays a pivotal role in the development of metabolic disorders. However, unlike host metabolic reprogramming, which has been well characterized, the pathogenic mechanisms resulting from gut microbiota metabolic reprogramming remain poorly understood, creating a critical knowledge gap regarding its role in systemic metabolic diseases. To address this gap, this review introduces the concept of gut microbiota metabolic reprogramming and establishes its foundational role in systemic metabolic disease. We propose that gut microbiota metabolic reprogramming constitutes an early pathogenic event, preceding and potentially driving subsequent metabolic alterations in the host. Within this framework, we systematically reveal that an imbalance in the gut microbiota leads to its significant metabolic reprogramming, including lipid, glucose, amino acid, and uric acid metabolism, which in turn regulates host-wide metabolic and immune homeostasis and contributes to the development of metabolic diseases. By integrating these mechanisms into a coherent model, our work provides a novel paradigm for understanding metabolic regulation. This model refines the fundamental pathophysiology of metabolic disorders and highlights new possibilities for targeting the microbiome for the prevention and treatment of metabolic disorders.

## Graphical abstract

**Figure:**
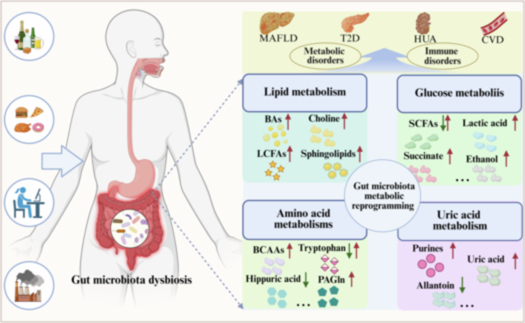

Long-term high-fat diets, alcohol abuse, and prolonged sedentary behavior can lead to gut microbiota dysbiosis, triggering gut microbiota lipids, glucose, amino acids, and uric acid metabolic reprogramming. This results in metabolic disorders and immune disorders, ultimately driving the onset of metabolic diseases. BAs: Bile acids; LCFAs: Long-chain fatty acids; BCAAs: Branched-chain amino acids; PAGln: Phenylacetylglutamine; SCFAs: Short-chain fatty acids; XOD: Xanthine oxidase; MAFLD: Metabolic dysfunction-associated fatty liver disease; T2D: Type 2 diabetes; HUA: Hyperuricemia; CVD: cardiovascular disease.

## 1. Introduction

The global epidemic of metabolic diseases, such as obesity, type 2 diabetes (T2D), and metabolic dysfunction-associated fatty liver disease (MAFLD), has emerged as a major public health challenge.^1^ Although current therapies have shown partial efficacy, patient heterogeneity and treatment resistance underscore the urgent need for novel therapeutic strategies.^2^ In recent years, the gut microbiota has gained widespread recognition as a “hidden organ” that regulates host metabolism.^3^ Made up of trillions of bacteria, fungi, and viruses, this microbial community exists in a complex symbiotic relationship with the human body.^4^ Research has indicated a strong association between microbial dysbiosis and the onset of metabolic disorders.^5^ However, a deeper understanding of the gut microbiota is still needed to develop new therapeutic strategies.

Host metabolic (pathway) reprogramming refers to systemic alterations in gene expression, protein translation, or metabolic networks within host cells or tissues.^6^ It represents adaptive physiological reprogramming of metabolic pathways, fluxes, and energy utilization patterns, a concept that has been well established in fields such as metabolism and immunology.^7^ Recent research has further revealed reciprocal host-microbe interactions. These interactions occur through a close relationship between the gut microbiota and host metabolism and physiology, wherein microbes influence host physiology, notably via their metabolic activities and derived products such as enzymes and metabolites, while the host in turn regulates microbial communities through mechanisms such as immune surveillance.^8^ Of particular significance are microbial metabolic effects, which actively participate in host metabolism.^9^ In particular, the gut microbiota systematically actively participates in and regulates the remodeling of the host’s metabolic pathways and energy utilization patterns through multilevel interactions such as product signaling, receptor activation, immune networks, and the neuroendocrine axis.^10^ However, a comprehensive model of metabolic regulation from the gut microbiota to the host is lacking.

Extending the concept of gut microbiota metabolic reprogramming provides a valuable and unifying perspective for integrating these multilayered functions into a more coherent understanding. Gut microbiota metabolic reprogramming refers to the situation in which external environmental interference or an internal ecological imbalance leads to dysbiosis of the gut microbiota and changes in its metabolites and subsequently triggers pathological fluctuations in gut metabolic pathways, gradually causing the host to deviate from physiological homeostasis. Such reprogramming involves not only the metabolic division of labor and collaboration among different microbial members within the community but also the generation of bioactive metabolites, which act as critical effector and signaling molecules that directly or indirectly influence host physiological processes.^11^ It is now well known that the gut microbiota critically regulates the metabolism of lipids, glucose, amino acids, and uric acid in the gut, thereby profoundly influencing the development of host metabolic diseases.^12-15^ However, investigations into metabolic reprogramming have predominantly focused on host peripheral tissues, including the liver, adipose tissue, and muscle.^16^^,^^17^ This focus has left a key knowledge gap in which the specific and crucial role of gut microbiota metabolic reprogramming in driving systemic metabolic diseases remains to be elucidated.

To address this knowledge gap, this study introduces the concept of gut microbiota metabolic reprogramming and establishes its foundational role in systemic metabolic disease. We propose that this microbial reprogramming constitutes an early pathogenic event, preceding and potentially driving subsequent host metabolic alterations. Within this framework, we systematically elucidate how microbial metabolites function as key signaling molecules, including specific lipid, glucose, amino acid, and uric acid, to coordinate host-wide metabolic and immune homeostasis. By integrating these mechanisms into a coherent model, our work provides a novel paradigm for understanding metabolic regulation by shifting the focus toward gut-initiated pathways. This refines the fundamental pathophysiology of metabolic disorders and highlights new possibilities for targeting the microbiome for the prevention and treatment of metabolic disorders.

## 2. Metabolic reprogramming

### 2.1. Host metabolic reprogramming

Metabolic reprogramming refers to the systematic, active, and plastic restructuring of metabolic networks in cells or biological systems to adapt to external environmental stimuli or internal physiological demands.^18^ This process goes beyond fine-tuning basal metabolic homeostasis that involves the redistribution of flux, efficiency, and flow in core metabolic pathways such as glycolysis, oxidative phosphorylation, fatty acid (FA) oxidation/synthesis, and amino acid metabolism. The primary goal of metabolic reprogramming is to optimize resource allocation (energy production, biosynthetic precursors, and signaling molecules) to prioritize essential resources under specific conditions, forming the fundamental mechanism of biological adaptability and functional plasticity.^17^ Metabolic reprogramming was first proposed in cancer research as a theoretical framework explaining the hallmark shifts in cancer cell metabolism to meet their needs for abnormal proliferation.^19^ With in-depth research, the concept of metabolic reprogramming has been extended to include immunity, aging and microbiology.^17^^,^^20^^,^^21^

On the basis of its temporal characteristics, metabolic reprogramming can be categorized as acute (via rapid enzymatic-mediated regulation) or chronic (via changes in gene expression or structural remodeling). For instance, during the acute inflammatory phase, macrophages rely on glycolysis to produce ATP, which manifests as a “Warburg-like” rapid energy supply.^22^ MYC proto-oncogene (c-Myc) regulates amino acid transporters through transcription, triggering sustained metabolic reprogramming, which reflects chronic adaptation at the genetic level.^23^ According to the dominant direction, metabolic reprogramming can be divided into an energy priority type (concentrated production capacity pathway) and a synthesis priority type (focusing on material synthesis). For instance, tumor cells rapidly proliferate by increasing glycolysis activity and FA oxidation to meet their energy demands, establishing an “energy-priority” metabolic pattern.^24^ DeBerardinis's review indicated that Myc-driven metabolic network reprogramming primarily serves to meet biosynthetic requirements.^25^ In terms of stress type, reprogramming can be classified as hypoxia-induced or nutrient-restricted. For instance, studies on the tumor microenvironment have demonstrated that hypoxia serves as the core stimulus driving metabolic reprogramming, leading to increased glucose uptake and lactate production.^26^ Research on sepsis-associated acute kidney injury has indicated that lipopolysaccharide (LPS)-induced nutritional stress results in an imbalance in amino acid metabolism, thereby triggering metabolic reprogramming.^27^ Each reprogramming type demonstrates pathway-specific selectivity on the basis of variations in environmental stress. The body initiates metabolic reprogramming when facing environmental stressors or physiological demands to maintain homeostasis or adapt and survive under new conditions.^28^^,^^29^

### 2.2. Gut microbiota metabolic reprogramming

As the “first-pass barrier” for the metabolism of exogenous substances, the gut microbiota plays a pivotal role in host nutrition and toxin processing.^30^ Dietary components (e.g., FAs, amino acids, and plant polysaccharides) and environmental chemicals ingested by the host are first exposed to the metabolic network of the gut microbiota.^31^ The functions of the gut microbiota differ between healthy individuals and those with diseases. Under physiological conditions, the commensal microbiota generates bioactive molecules that maintain metabolic homeostasis through collaborative fermentation, transformation, or degradation. For instance, dietary fiber is fermented into butyrate, which exerts anti-inflammatory effects, and tryptophan can be metabolized into indolepropionic acid, which provides antioxidant protection.^32^ In contrast, under disease conditions, pathogenic bacteria compete for nutrient substrates, redirecting glucose metabolism toward lactate production rather than short-chain fatty acids (SCFAs) synthesis; the gut microbiota may also metabolize fats into secondary bile acids (BAs), thereby regulating inflammation.^33^ At this stage, the metabolic functions of the gut microbiota undergo significant alterations, manifested as pathological changes in metabolic pathways, efficiency, and end product profiles.

The core mechanism of gut microbiota metabolic reprogramming described here manifests primarily as pathological reprogramming of gut lipid, glucose, amino acid, and uric acid metabolic pathways. When the host engages in unhealthy lifestyle habits, consumes a high-fat/high-protein diet, encounters circadian rhythm disruption, smokes, or consumes excessive amounts of alcohol, dysbiosis of the host gut microbiota can result.^34^ In terms of gut microbiota lipid metabolic reprogramming, microbiota dysbiosis dysregulates host digestible substrates by altering pathways such as cholesterol metabolism, FA metabolism, choline metabolism, and sphingolipid metabolism. A typical manifestation is metabolic efficiency for substrates such as BAs, and ceramides that differs from that under physiological homeostasis, thereby promoting hepatic lipid deposition, inflammation, and insulin resistance, which exacerbates diseases such as obesity, MAFLD, and atherosclerosis.^35^ For instance, in high-fat diet-fed mouse models, *Fusimonas intestini* (*F. intestini*) drives fat accumulation by increasing intestinal palmitate levels, whereas a reduction in *Bacteroides* spp. abundance diminishes the production of anti-inflammatory lipids (e.g., arachidonic acid).^36^^,^^37^ Gut microbiota glucose metabolic reprogramming involves primarily pathological changes in microbial selection and abnormalities in the efficiency of carbohydrate metabolic pathways.^38^ Gut microbiota dysbiosis contributes to metabolic disorders through the dysregulation of key metabolites like SCFAs, and lactate. The underlying mechanisms involve several pathways, notably the G protein-coupled receptor 41 (GPR41)/glucagon-like peptide-1 (GLP-1) signaling pathway, AMPK signaling, and the lactate cycle pathway. In terms of gut microbiota amino acid metabolic reprogramming, dysbiosis of the microbiota modulates metabolism by altering pathways such as tryptophan metabolism, tyrosine metabolism, and aromatic metabolism.^39^ Gut microbiota amino acid metabolic reprogramming profoundly influences host metabolism and immune homeostasis through a multiorgan, multipathway network. This imbalance not only directly contributes to metabolic disorders such as obesity, diabetes, and hyperuricemia but is also closely associated with inflammation, neurodegeneration, cardiovascular diseases, and tumors.^40^^,^^41^ Furthermore, gut microbiota uric acid metabolic reprogramming primarily induces hyperuricemia by affecting uric acid production and excretion. A metagenomic analysis revealed a significant reduction in the abundance of uric acid-degrading bacteria (e.g., Lachnospiraceae), leading to the accumulation of uric acid precursors.^42^ Furthermore, in the pathogenesis of metabolic diseases (such as obesity, T2D, and MAFLD), gut microbiota metabolic reprogramming serve as critical links between poor dietary habits and systemic chronic low-grade inflammation. The key mechanism by which this process predisposes the body to metabolic diseases is that specific pathological conditions lead to the formation of a “metabolic disease-inducing” microbial metabolite profile, which subsequently drives the immune system to shift from “homeostasis maintenance” mode to “inflammatory destruction” mode.

The reprogramming of lipid, glucose, amino acid, and uric acid metabolism in the gut microbiota serves as the primary regulatory mechanism enabling gut microbes to adapt to physiological and pathological conditions. This metabolic reprogramming not only results from the initiation of disease but also acts as the core driver of the persistent deterioration characteristic of metabolic disorders. Gut microbiota metabolic reprogramming engages host-gut crosstalk, serving as a critical bridge connecting adverse environmental stimuli with metabolic diseases. Next, we explore this field, aiming to enhance our understanding of life sciences and provide assistance for disease diagnosis and treatment.

## 3. Gut microbiota metabolic reprogramming affects the occurrence of metabolic diseases in the host

The gut microbiome, which has been labeled the human “second genome”, plays a pivotal role in the onset of host metabolic diseases through its induction of gut microbiota metabolic reprogramming.^43^ A healthy gut microbiota plays vital roles in multiple aspects, including assisting in food digestion, particularly by breaking down complex carbohydrates such as cellulose, and strengthening the intestinal barrier by regulating the immune system, thereby maintaining immune and gut homeostasis in the host.^44^ However, this microbial equilibrium is highly susceptible to external disruptions. Extensive evidence has indicated that prolonged unhealthy lifestyles, including the consumption of high-fat/sugar diets, sedentary habits, circadian rhythm disruption, and chronic psychological stress, can significantly alter the gut microenvironment.^45-48^ These factors disrupt the balance of the gut microbiota, leading to reduced diversity, a decreased abundance of beneficial bacteria, and an increased abundance of harmful microbes. Such dysbiosis triggers dramatic changes in metabolites, including the production of beneficial SCFAs decreases, whereas the production of harmful substances such as endotoxin/LPS, oxidized trimethylamine *n*-oxide (TMAO), and proinflammatory aromatic compounds increases, heightening the body's susceptibility to disease.^49-51^ Therefore, modulating the host’s gut microbiome may represent a promising therapeutic approach for combating metabolic diseases. This review focuses on four key aspects of gut microbiota metabolic reprogramming, namely, that of lipids, glucose, amino acids, and uric acid, and aims to provide a new theoretical basis and research perspectives for intervention strategies to treat related diseases.

### 3.1. Gut microbiota lipid metabolic reprogramming drives host metabolic disorders

**Figure 1.**
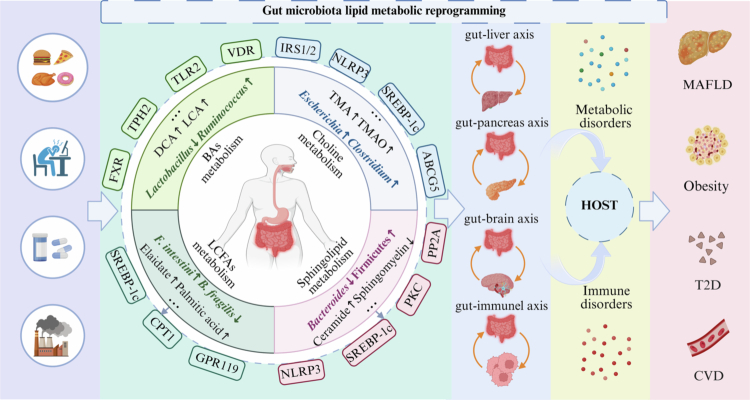
Gut microbiota lipid metabolic reprogramming affects host lipid metabolism, glucose metabolism, amino acid metabolism and immune regulation, thereby driving the development of metabolic diseases. BAs: Bile acids; LCFAs: Long-chain fatty acids; DCA: Deoxycholic acid; TMAO: Trimethylamine *n*-oxide; FXR: Farnesol X receptor; TPH2: Tryptophan hydroxylase 2; TLR2: Toll like receptors 2; VDR: Vitamin D receptor; IRS1/2: Insulin receptor substrates 1/2; NLRP3: NOD-like receptor thermal protein domain associated protein 3; SREBP-1c: Sterol regulatory element-binding protein-1c; ABCG5: Adenosine triphosphate- binding transporter G superfamily member 5; CPT1: Carnitine palmitoyltransferase; GPR: Glucose-dependent insulinotropic receptor; PP2A: Protein phosphatase 2 A; PKC: Protein Kinase C; PPAR: Peroxisome proliferator-activated receptor; MAFLD: Metabolic dysfunction-associated fatty liver disease; T2D: Type 2 diabetes; CVD: Cardiovascular disease; *F. intestini: Fusimonas intestini*; *B. fragilis: Bacteroides fragilis.*

Gut microbiota lipid metabolic reprogramming refers to a critical biological phenomenon in which the gut microbiota precisely regulates gut lipid metabolic pathways, thereby influencing the maintenance of physiological homeostasis and the progression of pathological processes in the host. During this process, the gut microbiota does not function in isolation but participates in the precise regulation of host metabolism through multilevel and multidimensional dynamic interactions.^52^ These interactions create a “microbiota-host cometabolic network” that is highly coordinated with host metabolic pathways. By affecting core pathways such as the cholesterol, FA, choline, and sphingolipid pathways, these interactions not only directly regulate lipid metabolism homeostasis but also indirectly influence glucose, amino acid, and uric acid metabolism through cross-regulatory interactions within the metabolic network.^53^ Overall, gut microbiota lipid metabolic reprogramming plays a significant role in the development of diseases of metabolism, including obesity, nonalcoholic fatty liver disease, and cardiovascular disorders (Figure 1).

#### 3.1.1. Bile acids (BAs)

##### 3.1.1.1. BAs regulate the synthesis and accumulation of lipids.

Gut BA metabolic reprogramming plays a critical role in host lipid metabolism. Under physiological conditions, the gut microbiota maintains lipid homeostasis through host-related metabolism. For instance, in healthy mice, *Lactobacillus reuteri* GG can degrade cholesterol to produce BAs and regulate host metabolism by activating the farnesol X receptor (FXR), forming a “microbiota-BA-host” regulatory axis.^54^ However, unhealthy lifestyle factors such as an irregular diet, smoking, and alcohol consumption disrupt the gut microbiota balance, triggering the metabolic reprogramming of BAs. This process leads to a significant elevation in and an abnormal composition of BAs in the intestinal lumen, particularly manifesting as a severe imbalance between primary and secondary BAs, with markedly increased levels of taurocholic acid, deoxycholic acid (DCA), and *ω*-muricholic acid.^55^ This abnormal BA composition interferes with FXR function and expression, resulting in aberrant cholesterol 7α-hydroxylase (CYP7A1) expression and exacerbating disorders of BA synthesis. Meanwhile, low expression of FXR upregulates the expression of sterol regulatory element-binding protein-1c (SREBP-1c) through an SHP-dependent mechanism, which in turn increases the expression of stearoyl-CoA desaturase 1 (SCD1), fatty acid synthase (FAS), and acetyl-CoA carboxylase (ACC), ultimately leading to increased hepatic lipid synthesis.^56^^,^^57^ Low FXR expression reduces the expression of Peroxisome proliferator-activated receptor *α* (PPARα) and carnitine palmitoyltransferase 1 (CPT1) to decrease FA oxidation, thereby reducing fat consumption and resulting in hepatic lipid accumulation.^58^ Low FXR expression induces the expression of scavenger receptor cluster of differentiation 36 (CD36), which increases FA uptake and directly regulates apolipoprotein C-II to reduce triglyceride (TG) hydrolysis, leading to abnormal hepatic lipid metabolism. These ultimately accelerates the progression of MAFLD.^59^ Studies on the effects of germ-free and conventional feeding of wild-type Fxr^-/-^ mice revealed that the gut microbiota promotes weight gain and hepatic steatosis by altering the BA composition and reducing hepatic pool volume through FXR signaling.^60^

##### 3.1.1.2. BAs regulate glucose metabolic homeostasis and insulin secretion through FXR/TGR5 signaling.

Gut BAs not only serve as core mediators in cholesterol metabolism but also participate in the regulation of systemic glucose homeostasis through FXR.^61^ Under physiological conditions, a dynamic balance is maintained among BA synthesis in the liver, BA enterohepatic circulation, and BA transformation by the gut microbiota. The long-term consumption of high-sugar, high-calorie diets significantly alters the gut microbiota structure.^62^^,^^63^ Dysbiosis of the gut microbiota can trigger metabolic reprogramming of BAs. When the abundance of the phylum Bacteroidetes is reduced and that of the phylum Firmicutes (particularly pathogenic or overgrown strains) increases, the overall relative activity of CYP7A1 is increased, leading to the conversion of more primary bile acids into DCA and lithocholic acid (LCA). These secondary bile acids subsequently exhibit abnormal accumulation in the intestine.^64^^,^^65^ Compared with primary BAs, secondary BAs exhibit weaker affinity for FXR, significantly attenuating gut FXR signaling.^66^ This results in reduced secretion of the intestinal hormones FGF15/19, which may weaken their nutritional and supportive effects on pancreatic *β*-cells, which is considered one of the mechanisms leading to insulin secretion dysfunction. Moreover, reduced FGF15/19 secretion indirectly affects GLP-1 secretion, exacerbating insulin resistance.^67^ Additionally, elevated levels of secondary BAs increase G protein-coupled bile acid receptor 1 (TGR5) agonism. TGR5 activation, particularly in intestinal L cells, leads to excessive GLP-1 secretion, which induces a compensatory increase in insulin secretion aimed at overcoming early insulin resistance. However, with long-term abnormal accumulation of secondary BAs, the negative feedback inhibition of FXR gradually becomes dominant, resulting in decreased GLP-1 secretion and pancreatic *β*-cell insulin secretion.^68^ Decreased insulin secretion induces a series of metabolic disorders in glucose metabolism. Owing to the lack of insulin-mediated glucose transporter (GLUT) activation on the cell membrane, glucose cannot enter cells for oxidative phosphorylation, leading to the accumulation of glucose in the blood and elevated blood glucose levels. Furthermore, as cells cannot obtain sufficient amounts of glucose for storage, glycogen synthesis is impaired, resulting in the inability of the liver to store sugar properly.^69^ Increased concentrations of free glucose in the blood trigger symptoms of metabolic diseases such as diabetes. Furthermore, excessively high levels of DCA/LCA may damage intestinal barrier function, induce low-grade inflammation (as metabolic endotoxins), and further injure pancreatic *β*-cells. Overall, alterations in bile acid composition disrupt glucose regulation by concurrently impairing FXR-mediated signaling and causing the overactivation of TGR5-mediated signaling.

##### 3.1.1.3. BAs mediate communication in the gut-brain axis through the tryptophan-5-HT metabolic pathway.

Under normal physiological conditions, the gut microbiota communicates bidirectionally with the central nervous system through the gut-brain axis. Specific gut bacteria, such as *Akkermansia muciniphila* (*A. muciniphila*)*, c*an regulate host BA metabolism via bile salt hydrolase (BSH) activity. This may lead to alterations in the levels of BA metabolites, including secondary BAs (e.g., DCA) and conjugated BAs (e.g., taurocholic acid). Animal studies have demonstrated that BA signaling may indirectly modulate the expression or activity of tryptophan hydroxylase 2 (TPH2), a key rate-limiting enzyme for 5-HT synthesis in the central nervous system, in the raphe nucleus of the brainstem.^70^ In the context of mental disorders such as depression, dysbiosis of the gut microbiota (including changes in *A. muciniphila* abundance), disturbances in BA metabolism, and abnormal levels of secondary BAs are frequently observed, leading to deviations in tryptophan metabolism, which may impair 5-HT neurotransmission in brain regions such as the hippocampus. Dysbiosis of the gut microbiota increases intestinal permeability, promoting the secretion of LPS and proinflammatory cytokines (e.g., tumor necrosis factor-*α* [TNF-*α*]) and interleukin-1β [IL-1β]) into the circulation, thereby compromising the integrity of the blood-brain barrier. This ultimately results in a vicious cycle of chronic low-grade inflammation and neurotransmitter imbalance, impairing host physiological status and promoting disease onset and progression.^71^ Studies have shown that supplementation with *A. muciniphila* can effectively alleviate lipid metabolism disorders and cognitive dysfunction.^70^

##### 3.1.1.4. BAs participate in intestinal immune homeostasis by regulating macrophage polarization and T-cell function.

In terms of immunomodulation, BAs play a complex dual role, with their ultimate proinflammatory or anti-inflammatory effects depending on the specific BA type, local concentration, and tissue microenvironment. This antagonistic property is clearly demonstrated by the metabolic activities of bacteria of the same genus. For instance, under metabolic stress, such as high-fat diet consumption, the proliferation of certain *Clostridium* species in the gut alters the metabolic profile of BAs, which can trigger BA metabolic reprogramming. On the one hand, an increased abundance of *Clostridium* can lead to elevated levels of secondary BAs, such as DCA. DCA drives macrophages to polarize toward the proinflammatory M1 phenotype by activating toll-like receptor 2 (TLR2) on macrophages and triggering nuclear factor κB (NF-κB) signaling, thereby exacerbating colonic inflammation. The eradication of *Clostridium* species using vancomycin effectively reduces DCA levels and alleviates inflammatory infiltration.^72^ On the other hand, other bacteria within the same genus (e.g., *Clostridium scindens*) can convert primary BAs into different secondary metabolites, such as 3-oxo-LCA.^73^ 3-Oxo-LCA serves as an endogenous ligand for the vitamin D receptor (VDR), and activation of the VDR pathway promotes the differentiation and function of regulatory T cells in the colon, increasing mucosal immune tolerance and thereby reducing susceptibility to experimental colitis.^74^^,^^75^ Therefore, BAs play a dual role in the immune network, with their ultimate proinflammatory or anti-inflammatory effect depending on the delicate balance of specific molecules and receptors and the pathophysiological context.

In summary, BAs serve as critical signaling molecules and metabolic mediators, occupying a central position in the gut microbiota-host dialog. Their balance directly regulates metabolic, neurological, and immune homeostasis in the host, while BA disorder may drive the development of various chronic diseases.

#### 3.1.2. Choline

##### 3.1.2.1. Choline regulates lipid metabolism by affecting de novo fatty acid (FA) synthesis, and lipid output.

The gut microbiota metabolizes dietary substrates, including phosphatidylcholine, choline, L-carnitine, and betaine, to produce trimethylamine (TMA), which is subsequently oxidized to TMAO in the liver by flavin monooxygenases (FMOs).^76^ TMAO, an enterogenous uremic toxin, is closely associated with the pathogenesis and progression of various chronic diseases, including cardiovascular diseases, chronic kidney disease (CKD), and MAFLD. Factors such as the consumption of a high-fat diet can lead to gut microbiota dysbiosis, triggering the metabolic reprogramming of choline, which increases the abundance of microbiota capable of producing TMA (e.g., certain *Clostridium* and *Escherichia* species carrying the cutC gene), thereby increasing the abundance of TMAO in the host.^7^^,^^77^ TMAO can drive lipid metabolism disorders through interconnected mechanisms at multiple levels. First, it upregulates the expression of SREBP-1c, thereby facilitating de novo FA synthesis.^78^^,^^79^ Second, it further increases hepatic lipid synthesis by upregulating the expression of hydroxymethylglutaryl-CoA synthase 1 (HMGCS1).^80^ Third, it regulates cholesterol transport by upregulating adenosine triphosphate-binding transporter G superfamily member 5/8 (ABCG5/8) while simultaneously downregulating Niemann-Pick c1-like protein 1 (NPC1L1) to inhibit reverse cholesterol transport, collectively leading to reduced hepatic lipid output and increased hepatic lipid accumulation.^81^^,^^82^ Additionally, TMAO can induce adipose tissue inflammation and exacerbate insulin resistance in adipocytes. Notably, the detrimental effects of TMAO extend beyond direct regulation of metabolic organs to the intestinal barrier and liver repair capacity. Studies have shown that TMAO downregulates the expression of zonula occludens-1 and occludin in the mouse colon and suppresses the Wnt/β-catenin signaling pathway, which is involved in tissue repair and metabolic homeostasis.^83^ This interference with the intestinal barrier and repair pathways may indirectly impair hepatocyte proliferation and repair capacity, disrupt normal metabolic programming, and increase the susceptibility of the liver to inflammatory stress and injury in the presence of lipid accumulation, thereby comprehensively driving and exacerbating hepatic lipid metabolism disorders and the progression of MAFLD.

##### 3.1.2.2. Choline influences glucose metabolism through modifications in insulin signaling and hepatic glucose production.

The gut microbiota-derived metabolite TMA and its hepatic oxidation product TMAO have been demonstrated to be closely associated with host glucose metabolism disorders. Under metabolic stress conditions, such as the consumption of a high-fat diet, gut microbiota dysbiosis triggers metabolic reprogramming of choline, thereby increasing the host TMAO load.^84^^,^^85^ TMAO inhibits the tyrosine phosphorylation of insulin receptor substrate 1/2 (IRS1/2) in insulin-sensitive tissues (e.g., the liver and muscle) and interferes with the activation of the PI3K/Akt signaling pathway, thereby impairing the metabolic regulatory function of insulin.^86^ In the liver, TMAO upregulates the expression of key gluconeogenic enzymes, such as phosphoenolpyruvate carboxykinase (PEPCK) and glucose-6-phosphatase (G6PC), thereby increasing gluconeogenesis, which exacerbates hyperglycemia under fasting or insulin-resistant conditions.^87^ After the addition of TMAO to the drinking water of mice fed a high-fat diet, fasting insulin levels and insulin resistance significantly increased, and the impairment of glucose tolerance induced by the high-fat diet was exacerbated.^88^ These findings indicate that TMAO serves as a critical metabolic hub linking gut microbiota dysbiosis to systemic insulin resistance, impaired glucose tolerance, and even an elevated risk of type 2 diabetes by disrupting insulin signaling and increasing hepatic glucose output.

##### 3.1.2.3. Choline induces branched-chain amino acids (BCAAs) accumulation, leading to a vicious cycle characteristic of metabolic disorders.

Under physiological conditions, TMAO functions as a crucial organic osmotic pressure regulator and molecular chaperone, maintaining protein stability and energy homeostasis under stress conditions.^89^ Studies suggest that TMAO may indirectly participate in metabolic regulation by influencing the activity of transcription factors associated with energy sensing. However, long-term high-fat diet-induced gut microbiota dysbiosis leads to pathological elevation of TMAO levels.^90^ At high concentrations, TMAO becomes a key driver of metabolic disorders. Under insulin-resistant conditions in the presence of elevated levels of TMAO, impaired amino acid utilization in peripheral tissues (particularly skeletal muscle) is frequently observed, resulting in abnormal accumulation of branched-chain amino acids (BCAAs) and aromatic amino acids (AAAs) in the blood.^91^ This excessive accumulation of BCAAs leads to the continuous activation of mTORC1 signaling, inhibiting the phosphorylation of IRS-1 and impeding downstream insulin signaling.^92^ BCAA accumulation not only serves as a marker of amino acid metabolic disorders but also may further exacerbate insulin resistance and metabolic disorders, creating a vicious cycle.

##### 3.1.2.4. Choline regulates systemic inflammation by affecting TLR4/NF-κB and NLRP3 inflammasome activity.

Dysbiosis of the gut microbiota leads to elevated TMAO levels, which can drive systemic inflammation through multiple mechanisms. One of the core pathways involves the activation of the TLR4/MyD88/NF-κB signaling pathway in immune cells (e.g., macrophages), promoting the production of proinflammatory factors such as TNF-*α*, IL-1β, and IL-6,^78^ as well as facilitating cholesterol accumulation and foam cell formation within macrophages.^7^ Another key mechanism is the activation of the NOD-like receptor heat protein domain-associated protein 3 (NLRP3) inflammasome, which subsequently activates caspase-1 and triggers a robust inflammatory response. This process not only directly induces vascular inflammation and endothelial dysfunction^93^ but also indirectly affects T-cell metabolism, increasing the production of interferon-*γ*, IL-*β* and TNF-*α* and thereby amplifying the inflammatory response.^94^ Experimental evidence has demonstrated that TMAO-induced M1 macrophage polarization and the expression of related proinflammatory markers (e.g., IL-1β, IL-6, and TNF-*α*) are significantly attenuated after treatment with NLRP3 inhibitors, highlighting the central role of this inflammasome.^95^ Thus, TMAO, as a multifunctional immune metabolic modulator, affects the trajectory of inflammatory and immune responses under various pathological conditions through the integration of innate and adaptive immunity.

##### 3.1.2.5. Choline modulates multiorgan signaling pathways implicated in hypertension, thrombosis, and tumor progression.

In healthy individuals, TMAO concentrations are tightly regulated at approximately 5.72 mM through the complex interplay of dietary intake, microbial composition, and host metabolism. However, various environmental and lifestyle factors can disrupt this balance. Prolonged exposure to a high-salt diet, excessive alcohol consumption, chronic mental stress, and insufficient physical activity induce gut microbiota dysbiosis, specifically increasing the abundance of TMAO-producing bacteria such as *Emergencia timonensis* and *Ihubacter massiliensis*. This dysbiosis leads to elevated TMAO levels, exacerbating angiotensin II-induced hypertension through the PERK/ROS/CaMKII/PLCβ3 axis and intensifying angiotensin II-mediated vasoconstriction.^96^ High TMAO levels promote cardiac fibrosis by activating signaling pathways that accelerate fibroblast-to-myofibroblast transformation, worsening postmyocardial infarction cardiac function. TMAO also increases platelet reactivity by promoting calcium ion release from intracellular stores, potentiating the inositol trisphosphate signaling pathway and increasing platelet aggregation in response to thrombin stimulation, which are key steps in thrombus formation.^97^ Furthermore, in patients who chronically consume moldy foods, abuse alcohol, or smoke, the quantity of the gut microbes associated with TMAO metabolism increases significantly, thus leading to elevated levels of TMAO. TMAO stimulates bone marrow-derived macrophages to secrete proinflammatory cytokines and promotes the proliferation and migration of pulmonary artery smooth muscle cells, resulting in pulmonary vascular remodeling and an increased risk of pulmonary hypertension.^98^ When TMAO enters the systemic circulation, it induces osteopontin expression in hepatocellular carcinoma cells under inflammatory conditions. This protein activates the ILK/AKT/mTOR signaling pathway, promoting cell proliferation, migration, and tumor microenvironment reprogramming, ultimately driving the malignant progression of liver cancer.^99^ Several studies have reported that inhibiting bacterial TMA synthesis effectively reduces TMAO synthesis and TMAO-induced atherosclerosis in mice. For instance, reducing intestinal bacterial production of TMA through the use of TMA lyase inhibitors can alleviate atherosclerosis and thrombotic events, suggesting that these molecules may be potential therapeutic targets for the treatment of cardiovascular disease patients.^100-102^

In conclusion, TMAO, as a key metabolite of the gut microbiota, is widely involved in pathological processes such as metabolic diseases, cardiovascular diseases, and tumors through its influence on multiple pathways, including inflammatory responses, insulin signaling, and lipid and amino acid metabolism. Inhibitors targeting bacterial TMA synthesis are promising novel therapeutic strategies for patients with these conditions.

#### 3.1.3. Long-chain fatty acids (LCFAs)

##### 3.1.3.1. LCFAs influence fat deposition and liver injury by regulating FA synthesis and *β*-oxidation.

As key environmental factors that regulate obesity, long-chain fatty acids (LCFAs) in the gut can drive fat deposition.^103^ Under normal physiological conditions, the stable structure of the gut microbiota maintains a dynamic equilibrium with host lipid metabolism.^104^ However, the consumption of high-sugar and high-fat diets significantly alters the microbial composition,^105^ indirectly triggering LCFA metabolic reprogramming. For instance, *F. intestini* colonization increases substantially in mice fed high-fat diets.^36^ *F. intestini* promotes the expression of FA synthesis gene clusters (e.g., fadR), which catalyze the extension of malonyl-CoA, significantly promoting the accumulation of elaidate and palmitic acid in the intestine. Palmitic acid is a potent activator of SREBP-1c, a transcription factor that upregulates genes such as those encoding FAS, thereby promoting de novo lipid synthesis in the liver, which drives weight gain and fat deposition.^36^ Notably, a typical Western diet (low in choline and high in fat and sugar) promotes the proliferation of *Blautia producta* (*B. producta*) in the mouse intestine. *B. producta* is a lipase-producing bacterium capable of generating 2-octagenoate (2-OG). 2-OG activates the transcription factors carbohydrate response element binding protein (ChREBP) and SREBP-1c, upregulating the expression of genes encoding enzymes involved in de novo lipogenesis and thereby facilitating the conversion of substrates (particularly glucose) into FAs and the synthesis of triglycerides in the liver. Meanwhile, 2-OG could inhibit CPT1 activity or expression to impede the entry of LCFAs into mitochondria, weakening mitochondrial *β*-oxidation and reducing the ability of the liver to degrade and utilize FAs. Consequently, 2-OG significantly increases lipogenesis and reduces FA oxidation.^106^

##### 3.1.3.2. LCFAs modulate the production of proinflammatory lipids and the release of inflammatory cytokines from immune cells.

Gut microbiota metabolic reprogramming not only directly regulates host lipid synthesis and degradation but also drives chronic inflammatory responses by inducing the production of specific proinflammatory lipid products, which is a critical step in the progression of metabolic diseases. For instance, upon consumption of a high-fat diet, in addition to its effects on hepatic lipid metabolism, the 2-OG produced by proliferating *B. producta* in the gut can accumulate in the liver and activate hepatic stellate cells and regulate macrophages through the GPR119/TAK1/NF-κB/TGF-β1 signaling pathway, thereby promoting hepatic fibrosis.^107^ Another common dietary pattern, high energy, low protein, disrupts microbial homeostasis, leading to a decrease in the abundance of beneficial bacteria (e.g., *Bacteroides*). Notably, the reduction in *Bacteroides fragilis* is closely associated with the disorder of host hepatic oxidative lipid metabolism.^108^ This disorder manifests at the molecular level as alterations in the expression or activity of key metabolic enzymes (e.g., arachidonic acid 15-lipoxygenase and 5-lipoxygenase), thereby reshaping the associated metabolite profile. Specifically, this metabolic reprogramming results in increased production of proinflammatory oxidized lipids (e.g., 15-oxo-ETE), while anti-inflammatory lipids or their precursors (e.g., free arachidonic acid) may be relatively depleted or imbalanced.^37^ These accumulated proinflammatory oxidized lipids (e.g., 15-oxo-ETE) can act as ligands to activate GPCRs on the surface of macrophages, neutrophils, and other cells, driving their production of inflammatory cytokines such as TNF-*α* and IL-1β and thereby exacerbating hepatic inflammatory responses.

Overall, LCFAs, as essential molecules in metabolism, play critical roles in maintaining energy balance, cellular membrane structure, and signal transduction. They not only serve as the primary energy source for cells but also regulate lipid metabolism and inflammatory responses through interactions with GPCRs and transcription factors.

#### 3.1.4. Sphingolipids

##### 3.1.4.1. Sphingolipids regulate the PPARα/SREBP-1c axis to modulate hepatic lipid metabolism.

Sphingolipid metabolic reprogramming plays a critical role in regulating host lipid homeostasis.^109^ In healthy individuals, the gut microbiota (particularly members of the phylum Bacteroidetes) can synthesize sphingolipids de novo. This process relies on microbial serine palmitoyltransferase (SPT) activity and a series of subsequent enzymatic reactions to generate various sphingolipids, including ceramide.^110^ These microbiota-derived sphingolipids can be absorbed by the host and, upon entering the liver, increase FA *β*-oxidation and mitochondrial respiratory chain efficiency by upregulating PPARα.^77^^,^^111^ However, when consuming a high-fat diet or receiving antibiotic treatment, sphingolipid metabolism is reprogrammed. Sphingolipid metabolism dysbiosis leads to a decrease in the abundance of microorganisms that synthesize beneficial sphingolipids (e.g., certain Bacteroidetes species), resulting in reduced SPT activity and decreased microbial sphingolipid output.^112^ This indirectly leads to insufficient ligand availability for PPARα activation in the host liver, leading to reduced activity of the FA *β*-oxidation pathway, decreased acylcarnitine production, and impaired oxidative FA degradation in mitochondria. Meanwhile, this also leads to the increment of SREBP-1c activity in the liver, promoting TG synthesis and ultimately resulting in hepatic lipid accumulation.^113^

##### 3.1.4.2. Sphingolipids influence insulin signaling, leading to alterations in glucose transport and utilization.

Considering the gut-liver axis, Bacteroidetes are the primary microbial sources of sphingolipid-synthesizing bacteria in vivo.^114^ A study by Johnson et al. demonstrated that Bacteroidetes (particularly *Bacteroides thetaiotaomicron*) can synthesize sphingolipids, which can enter the host via enterohepatic circulation, leading to a significant increase in the levels of odd-chain sphingolipids and ceramides in the liver.^115^ The long-term consumption of a high-fat diet decreases the abundance of beneficial symbiotic bacteria such as Bacteroidetes, while excessive dietary fat provides precursors for the synthesis of simple ceramides by other bacteria (e.g., certain members of the phylum Firmicutes). Disruption of the gut microbiota leads to ceramide metabolic reprogramming. Thus, the total amount and proportion of ceramides derived from microorganisms change, with more proinflammatory and insulin signaling-disrupting ceramide molecules being absorbed by the host. Ceramides are known inhibitors of the insulin signaling pathway and proinflammatory factors, and their accumulation triggers the reprogramming of signaling pathways within hepatocytes, as follows: 1) the activation of PKC ζ/λ inhibits the tyrosine phosphorylation of IRS, thereby blocking the downstream transmission of insulin signals;^116^ and 2) the activation of protein phosphatase 2 A (PP2A) leads to the dephosphorylation and inactivation of Akt, which directly prevents the translocation of GLUT4 to the cell membrane, resulting in impaired glucose uptake.^117^ This reduces tissue glucose uptake, exacerbates metabolic disorders, and results in a vicious cycle of “dysbiosis-sphingolipid abnormalities-insulin signaling blockade-glucose metabolism disorders”.^118^

##### 3.1.4.3. Sphingolipids contribute to local and systemic inflammation through the activation of the NLRP3 inflammasome.

Dysbiosis of the gut microbiota (including a reduction in *Bacteroides* abundance) leads to reduced synthesis of specific sphingolipids that may have signaling or structural functions. This alteration disrupts the lipid signaling homeostasis in the gut or systemic tissues, potentially indirectly triggering the compensatory upregulation of endogenous ceramide biosynthesis pathways in host tissues (e.g., the liver and adipose tissue). Ceramides act as potent activators of the NLRP3 inflammasome, promoting the cleavage and maturation of caspase-1, which drives the release of proinflammatory cytokines such as IL-1β and IL-18, exacerbating local and systemic inflammatory responses.^119^ This chronic inflammatory state not only further impairs insulin sensitivity but also is closely associated with hepatocyte injury and fibrosis. Additionally, ceramide accumulation induces endoplasmic reticulum stress and mitochondrial dysfunction, increasing the production of ROS. Through these mechanisms, ceramides play pivotal roles in metabolic disorders, inflammatory cascades, and lipid-mediated cytotoxicity.^120^

In summary, sphingolipids, a class of important bioactive lipids, are widely involved in various pathological processes, such as metabolic disorders, through the regulation of key pathways, including signal transduction, apoptosis, and inflammatory response pathways. Regulators targeting key metabolic enzymes (e.g., ceramidase or sphingosine-1-phosphate receptors) are emerging as novel therapeutic strategies for patients with these conditions.

Gut microbiota lipid metabolic reprogramming regulates host metabolism and immunity through multiple pathways, profoundly influencing the onset and progression of various diseases (Table 1). These findings highlight the importance of gut microbiota-derived metabolites as therapeutic targets. Future research should focus on exploring their molecular mechanisms and developing precision treatment strategies based on dietary modifications, microbiome engineering, or metabolite antagonists, thereby opening new avenues for managing metabolic disorders, cardiovascular diseases, and neuropsychiatric conditions.

**Table 1.** Key microbial metabolites, host receptors, and altered pathways in the context of gut microbiota lipid metabolic reprogramming.

Microbial metabolite(s)	Full name(s) of the metabolite(s)	Related beneficial bacteria	Related harmful bacteria	Host receptor(s)	Altered signaling or metabolic pathway(s)	Terminal effect on the host
Secondary BAs (e.g. DCA and LCA) and altered BA pool↑	Deoxycholic acid, lithocholic acid	*Lactobacillus reuteri GG↓*	/	FXR↓	BA synthesis↓ De novo lipogenesis↑ FA *β*-oxidation↓ FA uptake↑ TG hydrolysis↓	Lipid metabolism
		*Bacteroides↓ Prevotella↓*	*Clostridium*↑ *Helicobacter*↑ *Ruminococcus*↑	FXR↓	FGF15/19 secretion↓ GLP-1 secretion↓ Insulin secretion↓	Glucose metabolism
				TGR5↑	GLP-1 secretion↑ Chronic negative feedback via FXR-dominant GLP-1↓	
		*Akkermansia muciniphila↓*	/	/	5-HT synthesis*↓*	Amino acid metabolism
		*/*	*Clostridium*↑	TLR2↑	NF-κB signaling pathway↑ Proinflammatory M1 macrophage polarization↑	Immunomodulation
3-oxo-LCA↑	3-Oxo-lithocholic acid	*Clostridium scindens↑*	/	VDR↑	T-cell generation/function↑	
TMAO↑	Trimethylamine *n*-oxide	/	*Clostridium↑ Escherichia↑*	ABCG5/8↑ NPC1L1↓	De novo FA synthesis↑ Hepatic lipid synthesis↑ Hepatic lipid output↓	Lipid metabolism
				IRS1/2↓	PI3K/Akt signaling pathway↓ Gluconeogenesis↑	Glucose metabolism
				IRS1↓	mTORC1 signaling↓	Amino acid metabolism
				/	TLR4/MyD88/NF-κB signaling pathway↑ NLRP3 inflammasome↑	Immunomodulation
Elaidate and palmitic acid↑	/	/	*Fusimonas intestini*↑	/	FA synthesis↑	Lipid metabolism
2-OG↑	2-Octagenoate	/	*Blautia producta*↑	CPT1↓	FA synthesis↑ TG synthesis↑ *β*-oxidation↓	
				GPR119↑	TAK1/NF-κB/TGF-β1 signaling pathway↑	Immunomodulation
15-Oxo-ETE↑	/	*Bacteroides fragilis*↓	/	GPCRs↑	Inflammatory cytokines↑	
Sphingolipids↓	/	Bacteroidetes↓	/	/	FA *β*-oxidation↓ TG synthesis↑	Lipid metabolism
Ceramides↑	/	Bacteroidetes↓	Firmicutes↑	PKC ζ/λ↑ IRS1/2↓	Insulin signaling↓ Peripheral glucose uptake↓	Glucose metabolism
				/	NLRP3 inflammasome↑	Immunomodulation

### 3.2. Gut microbiota glucose metabolic reprogramming drives host metabolic disorders

**Figure 2.**
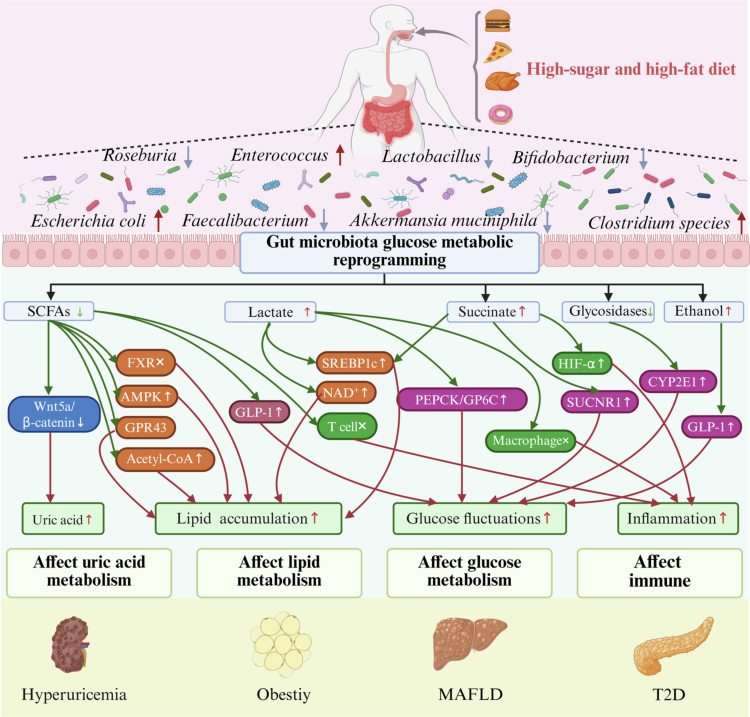
Gut microbiota glucose metabolic reprogramming affects host lipid metabolism, glucose metabolism, uric acid metabolism and immune regulation, thereby driving the development of metabolic diseases. SCFAs: Short-chain fatty acids; GPR43: G Protein-Coupled Receptor 43; SREBP1c: Sterol regulatory element-binding protein 1c; GLP-1: Glucagon-Like Peptide-1; FXR: Farnesol X receptor; PEPCK: Phosphoenolpyruvate carboxykinase; GP6C: Glucose-6-phosphatase catalytic; HIF-α: Hypoxic inducible factor-α; SUCNR1: Succinate Receptor 1; CYP2E1: Cytochrome P450 2E1; MAFLD: Metabolic dysfunction-associated fatty liver disease; T2D: Type 2 diabetes.

Reprogramming gut microbiota glucose metabolism refers to the systematic reprogramming of gut glycometabolic pathways, regulatory networks, and microbiota interactions under conditions of gut microbiota dysbiosis. This reprogramming influences lipid metabolism, amino acid metabolism, and glucose homeostasis by altering glucose absorption and utilization and the direction of energy metabolism flux (Figure 2).

#### 3.2.1. Short-chain fatty acids (SCFAs)

##### 3.2.1.1. SCFAs regulate lipid synthesis and catabolism.

SCFAs are a class of important metabolic products produced by gut microorganisms that are involved in the metabolism of dietary fiber. When undigested carbohydrates enter the intestines, the gut microbiota converts them into SCFAs, primarily acetate, propionate, and butyrate, through fermentation.^121^^,^^122^ Acetate is produced mainly by microorganisms such as *Bacteroides* and *Bifidobacterium*.^123^^,^^124^ Propionate is produced mainly by bacteria of the Lachnospiraceae and Negativicutes classes, as well as *Bacteroides* and *Akkermansia*.^125^ Butyrate is the main energy source for colonic epithelial cells and is produced by *Fusobacterium* spp., *Faecalibacterium*, *Eubacterium*, *Roseburia* and *Ruminococcus*.^126^^,^^127^

As a key external disrupting factor, a high-sugar diet has dual effects on the balance of the gut microbiota. On the one hand, it promotes the excessive production of acetate. Excess acetate enters the liver via the portal vein and acts as a key substrate for FA synthesis, facilitating de novo hepatic synthesis of FAs and TGs through the acetyl-CoA pathway.^128^ This process leads to hepatic lipid accumulation and increases the risk of MAFLD. On the other hand, acetate disrupts the overall balance of the gut microbiota, reducing the abundance of beneficial bacteria such as *Faecalibacterium* and *Roseburia*.^129^ Consequently, the levels of propionate and butyrate derived from the microbial metabolism of dietary polysaccharides decrease significantly, resulting in a series of adverse effects on host lipid metabolism and energy balance. First, the inhibitory effect of butyrate on hepatic cholesterol synthesis is weakened, and the regulatory function of this factor with respect to BA metabolism via the FXR-FGF15 signaling pathway is impaired, resulting in increased hepatic cholesterol synthesis, BA-mediated metabolic disorders, and potential increases in plasma cholesterol levels.^130^ Second, the weakened inhibition of histone deacetylases (HDACs) and the activation of AMPK by butyrate fail to effectively downregulate lipogenesis-related genes (e.g., SREBP-1c and FAS) while hindering the upregulation of lipolysis-related genes (e.g., hormone-sensitive lipase and adipose triglyceride lipase). This imbalance leads to increased fat synthesis, impaired fat decomposition, and increased fat accumulation in the liver and peripheral tissues.^131^ Additionally, the reduced ability of SCFAs to activate thermogenesis-related genes (e.g., uncoupling protein 1 [UCP1]) in brown adipose tissue and promote the browning of white adipose tissue via GPR43 signaling results in decreased oxidative lipid thermogenesis and energy expenditure, thereby exacerbating fat accumulation. Collectively, these changes induce systemic lipid metabolic disorders in the host and increase the risk of metabolic diseases such as obesity and MAFLD.^132^

##### 3.2.1.2. SCFAs influence insulin and glucagon secretion.

An imbalance in the structure of the gut microbiota further triggers the abnormal metabolism of SCFAs.^133^ The consumption of low-fiber industrial diets reduce microbial (*Faecalibacterium* and *Rothia*) production of SCFAs (especially butyrate), thereby disrupting gut-brain communication by weakening vagus nerve satiety signals and decreasing the secretion of satiety hormones such as CCK, GLP-1, and PYY. This leads to the brain misinterpreting the gut as empty.^134^ The resulting signal deficiency directly leads to insufficient insulin secretion and uncontrolled glucagon secretion, further exacerbating blood glucose fluctuations.

##### 3.2.1.3. SCFAs affect glutamate utilization and influence the nervous system.

In individuals experiencing chronic stress and high mental tension, abnormalities in the gut microbiota, such as an increase in the abundance of *Clostridium* species, lead to increased levels of their metabolic products, among which propionate may disrupt neuronal function by activating neuroinflammation, affecting the glutamate/γ-aminobutyric acid neurotransmitter system, or modulating HDAC activity, thereby exacerbating autism spectrum disorder-like behaviors.^135^

##### 3.2.1.4. SCFAs regulate host uric acid levels by modulating uric acid reabsorption and excretion.

When gut microbiota homeostasis is disturbed, the abundance of *Lactobacillus johnsonii, A. muciniphila* and *Lacticaseibacillus paracasei MJM60396* decreases, leading to insufficient production of metabolites such as butyrate. This causes ineffective activation of the Wnt5a/β-catenin signaling pathway, thereby downregulating the protein expression of ABCG2 in the gut and inhibiting the active excretion of uric acid into the gut lumen.^136^ Moreover, the expression of the uric acid reabsorption transporters urate transporter 1 (URAT1) and GLUT9 decreases owing to the reduced abundance of *A. muciniphila*, increasing the reabsorption of uric acid in the kidneys and further aggravating the accumulation of uric acid in the body.^137^

##### 3.2.1.5. SCFAs regulate T-cell function and influence inflammatory responses.

Gut microbiota SCFAs metabolic reprogramming regulates host metabolic pathways and plays a crucial role in maintaining intestinal immune homeostasis.^138^ When the gut microbiota becomes dysbiotic, SCFAs biosynthesis is often significantly reduced, and this deficiency directly affects intestinal immune balance.^139^ On the one hand, decreased SCFAs levels impair the generation and function of regulatory T cells, weakening immune suppression and tolerance mechanisms. On the other hand, microbiota dysbiosis drives the abnormal expansion of helper T17 cells and inhibits the generation of CD8⁺ memory T cells, leading to a proinflammatory immune response that activates inflammatory pathways such as the IL-17/IL-23 pathway.^140^^,^^141^ Animal studies have demonstrated that SCFAs supplementation can effectively increase the number of Tregs in the colonic lamina propria and reduce the proportion of proinflammatory Th17 cells, thereby alleviating experimental colitis.^142^ Overall, SCFAs deficiency caused by microbiota dysbiosis and the resulting intestinal immune dysbiosis are not confined to the local intestinal environment. This persistent low-grade inflammation can spread systemically, promoting chronic inflammation and significantly increasing the risk of metabolic diseases such as obesity, T2D, and MAFLD.

In summary, SCFAs, as key metabolites produced by the gut microbiota through the fermentation of dietary fiber, play pivotal roles in regulating intestinal homeostasis, metabolic disorders, and immune responses. This is achieved through multiple mechanisms, including the modulation of G protein-coupled receptor signaling, inhibition of histone deacetylase activity, and maintenance of intestinal barrier integrity. Dietary interventions aimed at promoting SCFA production or direct supplementation with specific SCFAs have emerged as critical strategies for disease prevention and treatment.

#### 3.2.2. Lactate

##### 3.2.2.1. Lactate regulates hepatic lipid synthesis and metabolism.

Lactate bacteria produce lactate mainly by decomposing sugars (monosaccharides, disaccharides, and oligosaccharides) and fermentable polysaccharides (starch and some dietary fibers).^143^ lactate is the end product of anaerobic metabolism and the main fermentation product of *Lactobacillus*, *Lactococcus* and *Bifidobacterium*.^144^ A high-sugar/high-carbohydrate diet provides abundant fermentation substrates for lactate-producing bacteria (including some nonprobiotic strains), leading to their overgrowth and the subsequent accumulation of lactate in the intestine. Excess lactate enters the liver via the portal vein and is converted into pyruvate, a precursor for gluconeogenesis, in hepatocytes and subsequently enters the citrate-pyruvate cycle as a substantial carbon source (acetyl-CoA) for de novo FA synthesis.^145^ Additionally, lactate itself acts as a signaling molecule, activating key transcription factors such as SREBP-1c and ChREBP and thereby upregulating the expression of lipid biosynthesis enzymes such as FAS and ACC.^146^ Moreover, the liver’s processing of excess lactate consumes cofactors such as NAD^+^, potentially inhibiting the mitochondrial *β*-oxidation of FAs and shifting the metabolic balance toward lipid synthesis. On the other hand, the accumulation of lactate in the intestine leads to a significant decrease in pH, creating an acidic environment that suppresses the growth of key beneficial bacteria such as butyrate-producing *Faecalibacterium*, thereby reducing the production of butyrate, which inhibits fat synthesis and promotes FA oxidation.^147^ A decrease in the content of SCFAs such as butyrate weakens their ability to activate the hepatic AMPK pathway and inhibit HDAC activity, thereby reducing their physiological regulation of lipid synthesis.^148^

##### 3.2.2.2. Lactate influences systemic insulin sensitivity through the modulation of gluconeogenesis and inflammation.

Excess lactate is converted into pyruvate, which significantly activates gluconeogenesis and upregulates the expression of PEPCK and G6PC, thereby abnormally increasing hepatic glucose output and directly elevating fasting and postprandial blood glucose levels.^149^ Furthermore, the accumulation of lactate in the intestine leads to an excessive decrease in the pH, inhibiting the growth of beneficial bacteria, including butyrate-producing bacteria such as *Faecalibacterium*. A decrease in the level of SCFAs such as butyrate decreases not only L-cell secretion of GLP-1 in the intestine but also the positive regulation of insulin signaling pathways in liver and peripheral tissues.^150^ Additionally, the lactate-induced acidic intestinal environment and dysbiosis damage the tight junctions of the intestinal epithelium, increasing endotoxins (e.g., LPS) entry into the circulation and thereby activating systemic low-grade inflammation.^151^ Inflammatory factors (e.g., TNF-*α* and IL-6) promote the serine phosphorylation of IRS by activating kinases such as JNK and IKKβ, inhibiting normal insulin signaling, and ultimately leading to insulin resistance in the liver, muscle, and adipose tissue.^152^ In this process, the glucose uptake and utilization capacity of muscle and adipose tissue decline, while hepatic glucose output continues to increase, collectively resulting in persistent hyperglycemia and further exacerbating systemic glucose metabolism disorders.

##### 3.2.2.3. Lactate regulates macrophage function to influence immune regulation.

Lactate plays complex and multifaceted roles in immune regulation. High concentrations of lactate can inhibit the glycolytic reprogramming of macrophages, weaken their response to microbial-associated molecular patterns such as flagellin, and reduce the production of proinflammatory factors such as IL-6 and IL-1β, thereby limiting their classical proinflammatory (M1-type) functions.^153^ Studies have revealed that in the tumor microenvironment, lactate drives the polarization of tumor-associated macrophages toward the M2 phenotype (anti-inflammatory/pro-fundus/protumor) through multiple mechanisms, including metabolic intervention, receptor signaling, and epigenetic regulation.^154^^,^^155^ These mechanisms not only elucidate how lactate shapes the immunosuppressive tumor microenvironment but also significantly increase its potential value as a biomarker for patient diagnosis or prognosis.

In conclusion, a diet-induced reduction in lactobacilli abundance and subsequent lactate metabolic disorders constitute critical factors that trigger a series of metabolic disorders, including intestinal dysbiosis, systemic inflammation, insulin resistance, and hepatic steatosis.

#### 3.2.3. Succinate

##### 3.2.3.1. Succinate influences hepatic lipid and cholesterol synthesis through the regulation of AMPK activity.

Succinate is an intermediate product of the tricarboxylic acid cycle that also serves as a key intermediate for propionate biosynthesis by the gut microbiota.^156^ Additionally, succinate is a fermentation product of certain symbiotic microorganisms (such as *Bacteroides* and *Prevotella*) that are metabolized collaboratively by both the microbiota and the host.^157^^,^^158^ Under pathological conditions such as obesity, alterations in microbial community structure not only manifest as changes in species abundance but also indicate a “reprogramming” of overall metabolic functions, characterized by an increased abundance of succinate-producing bacteria (e.g., Prevotellaceae and Veillonellaceae) and a decreased abundance of succinate-consuming bacteria (e.g., Odoribacteraceae and Clostridaceae). An imbalance in the abundances of these two microbial groups is a hallmark of metabolic disorders that directly leads to the abnormal accumulation of succinate, which originally functions in energy metabolism, and its transformation into pathological signaling molecules that disrupt host lipid homeostasis.^157^ High concentrations of succinate inhibit AMPK activity, leading to increased FA (via SREBP-1c) and cholesterol synthesis, thereby promoting the development of MAFLD and atherosclerosis.^159^

##### 3.2.3.2. Succinate contributes to insulin resistance through the modulation of SUCNR1-mediated inflammatory signaling.

Succinate accumulation is a potential risk factor for insulin resistance. Although it is not a direct insulin antagonist itself, succinate indirectly induces IRS-1 serine phosphorylation by activating host succinate receptor 1 (SUCNR1)-mediated inflammatory signaling (NF-κB, JNK, and MAPK), thereby suppressing the PI3K-AKT pathway and leading to decreased tissue sensitivity to insulin.^160^^,^^161^ The levels of inflammatory factors in the adipose tissue of SUCNR1-deficient mice were shown to be significantly reduced, suggesting that SUCNR1-mediated inflammatory signaling is a key factor in obesity-induced insulin resistance.^162^ DeVadder et al. utilized tracer studies to reveal that succinate, which acts as a substrate for gluconeogenesis in the intestine, can inhibit hepatic glucose output and significantly improve glucose metabolism.^157^ In summary, succinate plays a role in improving glucose metabolism.

##### 3.2.3.3. Succinate regulates the macrophage phenotype and intestinal immune response by stabilizing HIF-1α.

Dysbiosis of the gut microbiota often leads to the overgrowth of succinate-producing bacteria, resulting in the localized accumulation of succinate in the intestine.^157^ As a key metabolic intermediate and signaling molecule, succinate plays a complex and multifaceted role in immune regulation via hypoxia inducible factor-1α (HIF-1α). On the one hand, stable expression of HIF-1α promotes the generation of ROS and reactive nitrogen species (RNS), driving macrophages toward a proinflammatory M1 phenotype.^163^^,^^164^ On the other hand, the accumulation of HIF-1α significantly enhances glycolytic flux, and elevated peroxide levels further upregulate the expression of inflammation-related factors.^165^ In terms of gut-specific immunity, succinate regulates the function of intestinal tuft cells through its receptor GPR91, thereby promoting a Th2-type immune response.^166^ Additionally, the uptake of succinate by gut macrophages exacerbates inflammatory responses, which is consistent with clinical observations of elevated succinate levels in the intestines of patients with inflammatory bowel disease (IBD).^161^^,^^167^ However, the immunomodulatory effects of succinate are not unidirectional. Some studies suggest that succinate and its receptor GPR91 may also inhibit the inflammatory activation of macrophages, thereby modulating the pathogenesis of systemic diseases such as obesity.^168^^,^^169^ Recent studies have also revealed that microbe-derived succinate can restrict the migration of CD8⁺ T cells to the tumor microenvironment, thereby decreasing the efficacy of immune checkpoint inhibition for the treatment of CRC patients.^170^ Succinate is not merely a “proinflammatory molecule” but also a multifunctional and environment-dependent immune metabolic regulatory node. Its concentration in the local microenvironment, the types of cells it acts on, and the pathological context collectively determine the ultimate outcome of exacerbating inflammation, immune deviation, or suppressive regulation. This provides a critical perspective for understanding the complex mechanisms of microbiota-host interactions in metabolic and immune diseases.

In summary, the regulatory role of succinate in energy homeostasis is specific and closely associated with nutritional, physiological, and pathological conditions. A deeper understanding of the intrinsic molecular mechanisms underlying the multifunctional biological roles of succinate may provide novel targets and insights for the prevention and treatment of metabolic disorders.

#### 3.2.4. Glycosidase

Glycosidases (e.g., *β*-glucosidase) produced by the gut microbiota are key enzymes involved in the breakdown of dietary fiber.^171^ The consumption of a high-sugar, high-fat diet selectively promotes the overgrowth of certain pathogenic bacteria while inhibiting or reducing the activity of gut health-regulating bacteria.^172^ This imbalance indirectly leads to abnormalities in the types and activity levels of gut glycosidases. Overgrown pathogenic bacteria (e.g., *Escherichia coli* (*E. coil*) and *Enterococcus*) typically lack the enzymes (e.g., glycosidases) required to degrade complex dietary fibers and are thus unable to utilize these fibers as energy sources. Moreover, probiotics (e.g., *Bifidobacterium* and *Bacteroides*) exhibit reduced glycosidase activity, resulting in difficulty in degrading complex dietary fibers.^173^^,^^174^ Owing to the impaired degradation of complex fibers, nutrients in the gut (particularly carbohydrates) primarily exist in the form of monosaccharides (glucose and fructose). Pathogenic bacteria tend to utilize these simple sugars for rapid proliferation. The consumption of glucose by pathogenic bacteria stimulates intestinal epithelial cells to produce more “insulin secretagogues” (e.g., GLP-1), leading to excessive insulin secretion by the pancreas. This “insulin overshoot” causes a sharp initial increase in blood glucose levels (due to rapid glucose release into the bloodstream), followed by a rapid decline due to an excess of insulin, potentially even inducing hypoglycemia. This exacerbates postprandial glucose fluctuations, marking the initial stage of metabolic disorders.

#### 3.2.5. Alcohol

In patients consuming a high-fat diet, the abundance of alcohol-producing bacteria, including *Proteobacteria* and *Enterobacteriaceae*, increases. These bacteria can ferment unabsorbed glucose into ethanol, which enters the liver via the portal vein, inducing the activity of cytochrome P450 2E1 (CYP2E1). Increased CYP2E1 activity increases hepatocyte sensitivity to glucagon, promoting glycogenolysis and gluconeogenesis. Moreover, ethanol is metabolized by CYP2E1 to acetaldehyde, which directly inhibits Akt activity, reducing the translocation of GLUT4 to the cell membrane. Consequently, hepatocyte insulin sensitivity decreases, and glucose uptake by peripheral tissues (e.g., muscle and adipose tissue) decreases, exacerbating liver injury and glycometabolic disorders.^175^

In conclusion, the gut microbiota affects glucose metabolism in the body by altering glucose absorption, insulin sensitivity and gluconeogenesis through the regulation of the types and levels of glucose metabolites in the gut.

Gut microbiota glucose metabolic reprogramming is an adaptive process between the host and microbiota under environmental stress that is characterized by a shift in prioritized metabolic pathways and the rewiring of regulatory networks (Table 2). A deeper understanding of this process can provide novel targets for the prevention and treatment of metabolic diseases, such as achieving “beneficial reprogramming” of gut glycometabolism by modulating microbiota-derived metabolites and endocrine signaling.

**Table 2.** Key microbial metabolites, host receptors, and altered pathways in the context of gut microbiota glucose metabolic reprogramming.

Microbial metabolite	Full name of metabolites	Related beneficial bacteria	Related harmful bacteria	Host receptor(s)	Altered signaling or metabolic pathway(s)	Terminal effect on the host
Acetate↑	/	*Bacteroides*↑ *Bifidobacterium*↑	/		De novo FA synthesis↑ TG synthesis↑	Lipid metabolism
Butyrate↓	/	*Faecalibacterium*↓ *Roseburia*↓ *Rothia*↓	/	FXR↓ GPR43↓ UCP1↓	FXR-FGF15 signaling pathway↓ AMPK signaling pathway↓ Lipid oxidation↓	Lipid metabolism
	/		/	/	Vagal satiety signals↓ Satiety hormone (CCK, GLP-1, PYY) secretion↓ Insulin secretion↓	Glucose metabolism
	/	*Lactobacillus johnsonii*↓ *Akkermansia muciniphila*↓ *Lacticaseibacillus paracasei MJM60396*↓	/	ABCG2↓ URAT1↓ GLUT9↓	Wnt5a/β-catenin signaling pathway↓ Uric acid excretion↓ Uric acid accumulation↑	Uric acid metabolism
Propionate↑	/	*Clostridium species*↑	/	/	Glutamate/γ-aminobutyric acid neurotransmitter system signaling↑	Amino acid metabolism
SCFAs↓	Short-chain fatty acids	*Faecalibacterium*↓ *Roseburia*↓ *Rothia*↓	/	/	IL-17/IL-23 inflammatory pathways↑ T-cell generation/function↓	Immunomodulation
Lactate↑	/	*Lactobacillus*↑ *Lactococcus*↑	/	/	Citrate-pyruvate cycle↑ FA *β*-oxidation↓ AMPK signaling pathway↓	Lipid metabolism
		*Faecalibacterium*↓	/	IRS↓	Gluconeogenesis pathway↑ GLP-1 secretion↓ Insulin signaling↓ Glycolytic reprogramming↓	Glucose metabolism
		/	/	/	Macrophage glycolysis and response to MAMPs↓ Macrophage polarization toward an M2-like (protumor) phenotype↑	Immunomodulation
Succinate↑	/	Odoribacteraceae↓ Clostridaceae↓	Prevotellaceae↑ Veillonellaceae↑	/	AMPK signaling pathway↓ FA and cholesterol synthesis pathways↑	Lipid metabolism
				SUCNR1↑ IRS-1↓	PI3K-AKT insulin signaling↓ Glucose output↓	Glucose metabolism
				HIF-1α↑ GPR91↑	Proinflammatory cytokine secretion↑ Production in macrophages (M1 polarization)↑ Th2-type immune response↑	Immunomodulation
Glycosidase↓	/	*Bifidobacterium*↓ *Bacteroides*↓	*Escherichia coli*↑ *Enterococcus*↑	/	GLP-1 secretion↑	Glucose metabolism
Microbial Ethanol↑	/	/	*Proteobacteria*↑ *Enterobacteriaceae*↑	CYP2E1↑ Akt ↓ GLUT4↓	Gluconeogenesis↑ Glycogenolysis↑ Peripheral glucose uptake↓	Glucose metabolism

### 3.3. Gut microbiota amino acid metabolic reprogramming drives host metabolic disorders

**Figure 3.**
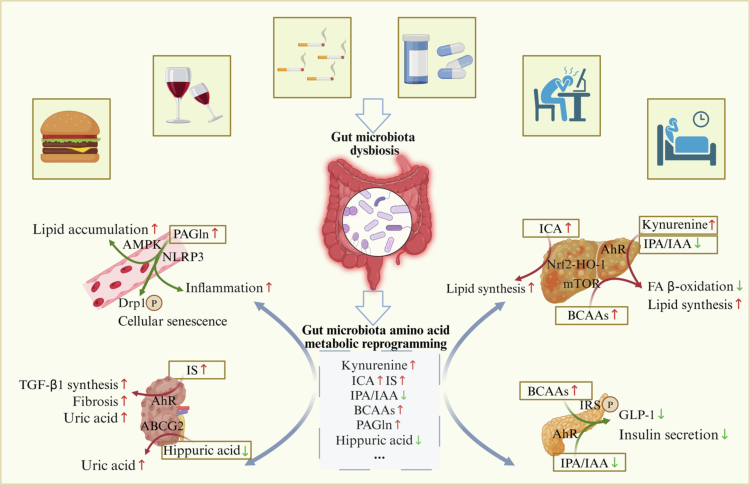
Gut microbiota amino acid metabolic reprogramming affects host lipid metabolism, glucose metabolism, uric acid metabolism and immune regulation, thereby driving the development of metabolic diseases. PAGln: Phenylacetylglutamine; AMPK: Adenosine 5‘-monophosphate(AMP)-activated protein kinase; NLRP3: NOD-like receptor thermal protein domain associated protein 3; ICA: Indole-3-carboxylic acid; IPA: 3-indolepropionic acid; IAA: Indole acetic acid; AhR: Aryl hydrocarbon receptor; mTOR: Mammalian Target of Rapamycin; FA: Fatty acid; IRS: Insulin receptor substrates; BCAAs: Branched-chain amino acids; GLP-1: Glucagon-Like Peptide-1; TGF-β: Transforming growth factor-β; IS: Indole sulfonate; ABCG2: Adenosine triphosphate-binding transporter G superfamily member 2.

Gut microbiota amino acid metabolic reprogramming represents an evolutionary strategy employed by microbial communities to adapt to dynamic changes in the internal environment of the host. Through the precise regulation of amino acid uptake, catabolism, anabolism, and interconversion, metabolic reprogramming ensures that the energy supply, signal transduction, and microenvironmental homeostasis are maintained. The dynamic equilibrium of this host-microbe cometabolic network is critical for health, and its disruption is closely associated with metabolic disorders, neurodegenerative diseases, and cancer (Figure 3).

#### 3.3.1. Tryptophan

##### 3.3.1.1. Tryptophan modulates hepatic lipid synthesis and degradation through regulation of the AhR pathway.

After entering the colon, tryptophan is converted by intestinal bacterial tryptophanases (tnaA, padA, fldA, etc.) into various metabolites, such as indole, tryptamine, indoleacetic acid (IAA], indolelactic acid (ILA), and 3-indolepropionic acid (IPA). The consumption of high-fat, high-fructose, and high-calorie diets, sedentary lifestyles, and excessive alcohol consumption significantly alter the characteristics of the gut microbiome, as the abundance of proinflammatory taxa such as *Proteobacteria*, Enterobacteriaceae, and *Escherichia* increases and the abundance of beneficial bacteria, including Ruminococcaceae and Rikenellaceae, decreases.^32^^,^^176^ This imbalance first disrupts tryptophan metabolic homeostasis, inducing tryptophan metabolic reprogramming, with abnormal activation of the kynurenine pathway being particularly critical. Kynurenine, a core metabolite in the gut microbiota-tryptophan-liver axis, is produced via indoleamine 2, 3-dioxygenase (IDO1) catalysis, and the upregulation of IDO1 is a hallmark of obesity.^39^ In patients with unhealthy habits, aberrant IDO1 activation leads to increased kynurenine production, which triggers obesity-related signaling by binding to aryl hydrocarbon receptor (AhR) and inhibiting PPARα and AMPK activity in hepatocytes, leading to the dephosphorylation and activation of ACC, promoting the production of malonyl-CoA, inhibiting FA *β*-oxidation, and increasing lipid synthesis, ultimately resulting in lipid accumulation within hepatocytes.^177^^,^^178^ Collectively, these phenomena affect hepatic lipid metabolism and promote the development of metabolic-associated hepatic steatosis (MHS) in nondiabetic adults.^39^ These findings suggest that tryptophan metabolites could serve as potential diagnostic biomarkers for MHS.

In another important branch of metabolism, tryptophan is metabolized by the gut microbiota to produce indole derivatives such as IPA and IAA, whose synthesis depends on the abundance of *Clostridium XIVa* and *Bifidobacterium*.^179^ Under normal physiological conditions, these bacteria generate IPA and IAA through tryptophan metabolism, activating the AhR signaling pathway to induce IL-22 expression.^180^ IL-22 not only improves intestinal barrier integrity to reduce LPS entry into the bloodstream but also activates hepatic lipid metabolic pathways, ameliorating hepatic steatosis by upregulating the expression of genes related to FA *β*-oxidation and downregulating the expression of genes related to lipid synthesis.^181^ Additionally, IPA and IAA reduce serum lipopolysaccharide-binding protein (LBP) levels, downregulate hepatic TLR4 expression, and inhibit NF-kB phosphorylation, thereby decreasing inflammatory cytokine release.^182^ However, the consumption of an unhealthy diet reduces the abundance of *Lactiplantibacillus* and *Bifidobacterium*, impairing the conversion of tryptophan to indole metabolites, which is characterized by decreased levels of IPA and IAA and tryptophan accumulation.^183^ HFD-fed mice exhibited significantly decreased AhR signaling activity, which can be attributed to the reduced production of AhR ligands, including indole, indole acetic acid, tryptamine, and 5-hydroxyindole acetic acid, by the gut microbiota through the indole metabolic pathway.^184^ Deficiencies in IPA and IAA directly weaken the protective effects of the AhR-AMPK-PPARα pathway. This metabolic reprogramming impairs the regulation of hepatic lipid synthesis and catabolism, exacerbates lipid metabolic imbalance and inflammatory responses, and promotes the progression of MAFLD.^185^^,^^186^ Treatment with FIZC (an AhR agonist) alleviated damage related to AhR signaling and improved metabolic disorders in HFD-fed mice.^184^ Furthermore, IPA affects the “gut-brain axis”, that after being secreted from the gut into the circulation, it directly binds to the src homology domain 2 of the signal transducer and activator of transcription 3 (STAT3) protein in the hypothalamus, promoting the phosphorylation and nuclear translocation of STAT3, increasing leptin sensitivity, and regulating the balance between appetite and energy metabolism. The long-term consumption of a high-fat diet reduces the abundance of gut *Clostridium* and the expression of the fldC gene, which encodes a key enzyme in IPA synthesis, leading to decreased IPA production, reduced STAT3 phosphorylation, impaired leptin sensitivity, and the exacerbation of lipid metabolic disorders.^41^ Studies have shown that IPA can improve high-fat diet-induced metabolic-related fatty liver disease by regulating adipose tissue metabolism.^40^ Through metagenomic studies, Lu et al. found that the abundance of Bacteroidaceae and Lactobacillaceae in the high-fat diet group was lower than that in the control group, and these bacteria have been proven to produce tryptase, which metabolizes tryptophan to produce indole-3-carboxylic acid (ICA).^187^ ICA deficiency prevents AhR activation and its subsequent binding to the nuclear factor erythroid 2-related factor 2 promoter (Nrf2), inactivating the Nrf2/HO-1 antioxidant pathway.^188^ This process leads to the accumulation of reactive oxygen species in vascular endothelial cells and increased expression of inflammatory factors, which constitute important triggers for atherosclerosis.^187^ These findings provide novel insights that can be used to target gut microbiota amino acid metabolic reprogramming for the prevention and treatment of MAFLD and atherosclerosis.

##### 3.3.1.2. Tryptophan contributes to insulin resistance by regulating GLP-1 secretion.

Tryptophan, an essential amino acid, is involved in protein synthesis and metabolic regulation, and its microbial metabolism is pivotal for glucose homeostasis.^189^^,^^190^ In healthy individuals, indole derivatives activate intestinal AhRs, inducing the expression of GLP-1, a key hormone that promotes insulin secretion from pancreatic *β*-cells, thereby improving glucose metabolism. These findings underscore the role of the microbiota in glucose regulation through amino acid metabolic pathways.^191^ However, unhealthy lifestyles that are characterized by overnutrition and physical inactivity lead to a significant reduction in gut microbiota diversity, with the abundance of tryptophan-metabolizing bacteria (e.g., *Lactobacillus* and *Rhodococcus*) potentially decreasing by 30-50%. Decreases in the abundance of beneficial bacteria and reduced indole-producing enzyme activity disrupt tryptophan (e.g., IPA) metabolic pathways, triggering the reprogramming of amino acid metabolism. Furthermore, increased tryptophan uptake by host tissues further depletes metabolic substrates, resulting in reduced indole metabolite production. Insufficient intestinal AhR activation then causes a 30%-40% reduction in GLP-1 expression in enteroendocrine L-cells, leading to insulin secretion defects.^192^ GLP-1 deficiency exacerbates *β*-cell dysfunction and insulin resistance, which in turn inhibits the proliferation of beneficial bacteria, resulting in the development of cyclic metabolic disorders that predisposes individuals to T2DM.^181^^,^^193^

##### 3.3.1.3. Tryptophan modulates the AhR pathway to influence renal injury progression and uric acid excretion.

An increasing number of studies have confirmed that AHR is a cellular receptor for toxins.^194^ Activation of AHR can lead to progressive damage to glomerular and tubular cells, subsequently triggering glomerulosclerosis and renal interstitial fibrosis, thereby exacerbating chronic kidney disease.^195^ A study on animals fed a high-fat protein diet revealed that animals fed a high-fat diet exhibited significantly elevated levels of indole and its metabolites (including indole sulfonate [IS]) in the cecum, colon, and feces.^196^ This substance is produced through hepatic sulfation of the microbial metabolite indole, which is converted from tryptophan via tryptophanase expressed by *E. coli* and *Typhamicrobacterium*. IS is considered toxic to the vasculature and contributes to renal dysfunction and cardiovascular diseases. IS can induce podocyte injury and glomerular damage through AHR activation. IS also induces the synthesis of TGF-β1, promoting glomerular fibrosis. Additionally, IS affects uric acid excretion, typically leading to elevated serum uric acid levels.^197^

##### 3.3.1.4. Tryptophan regulates immune cell differentiation and participates in intestinal inflammation and immunity.

The relationship between tryptophan metabolites and their receptor, AhR, is particularly significant in the context of the intestinal immune system. Dysbiosis of the gut microbiota disrupts tryptophan metabolism, leading to abnormal activation of the kynurenine pathway and resulting in elevated levels of kynurenine. Kynurenine strongly activates the expression of AhR and Kruppel-like factor 4. AhR not only inhibits the activation of NF-κB signaling but also upregulates the expression of the TAM exonuclease CD39, thereby promoting the dysfunction and exhaustion of CD8+ T cells.^198^ The long-term consumption of high-fat, high-sugar diets, circadian rhythm disruption, and a sedentary lifestyle can cause a gut microbiota imbalance, preventing the microbiota from producing AhR agonizts. These changes lead to insufficient AhR activation and reduced IL-22 secretion, ultimately triggering inflammation. Supplementation with *lactobacilli* capable of producing AhR agonizts can alleviate this inflammation. In summary, gut dysbiosis disrupts tryptophan metabolism, leading to aberrant kynurenine pathway activation and a subsequent imbalance in AhR signaling, which collectively drives inflammation.

Overall, tryptophan metabolites, as key products cometabolized by the gut microbiota and the host, are extensively involved in various pathological processes, such as immune-related diseases and metabolic syndrome. This is achieved through core mechanisms, including activation of aromatic hydrocarbon receptor signaling pathways, regulation of immune cell differentiation, and maintenance of intestinal barrier function.

#### 3.3.2. BCAAs

##### 3.3.2.1. BCAAs regulate lipid synthesis and deposition through the mTOR/SREBP-1c pathway.

BCAAs are derived primarily from protein-rich foods in the diet, such as meat, dairy products, legumes, and nuts.^14^ Studies have shown that individuals with poor lifestyle habits often exhibit an elevated Firmicutes to Bacteroidetes ratio in their gut. This alteration in microbial structure increases protein fermentation capacity in the gut, thereby increasing the release of free BCAAs and other amino acids. These excess BCAAs entering the circulation can activate mTOR signaling in the liver, upregulating the expression of the transcription factor SREBP1-c. Consequently, this promotes the expression of genes related to lipid synthesis, such as FAS and ACC, accelerating the synthesis of FAs and triglycerides.^199^ Moreover, BCAAs inhibit the expression of genes involved in PPARα-mediated FA oxidation, reducing lipid catabolism. The combined effects of increased lipid synthesis and decreased oxidation collectively contribute to the accumulation of lipids in the liver, which may increase the long-term risk of cardiovascular diseases.^200^

##### 3.3.2.2. BCAAs modulate the insulin signaling pathway to influence tissue glucose sensitivity.

The excessive intake of a high-fat, high-protein diet drives gut microbiota dysbiosis, characterized by the overgrowth of pathogenic bacteria, reduced abundance of *Bacteroides*, and increased abundance of *Prevotella*. This shift increases the quantity of BCAA precursors, triggering BCAA metabolic reprogramming and excessive BCAA production. High concentrations of BCAAs (e.g., leucine) bind to the mTOR complex, phosphorylating downstream S6 kinase 1 (S6K1) and inducing serine phosphorylation of IRS1, a modification that inhibits insulin signal transduction. This process impairs glucose uptake by the liver and muscles, reducing systemic insulin sensitivity. Additionally, high BCAA levels competitively inhibit insulin receptor phosphorylation and decrease GLUT4 translocation to cell membranes.^201^

##### 3.3.2.3. BCAAs regulate vascular cell function and modulate the progression of atherosclerosis.

BCAAs generated by *Parabacteroides merdae* can directly reduce the expression of mTORC1 downstream targets in plaques, reducing foam cell formation and inflammatory cascades. However, after the consumption of a high-fat diet, gut microbiota dysbiosis leads to decreased BCAA catabolism, elevated blood BCAA levels, activation of the mTORC1 pathway, the promotion of vascular smooth muscle cell proliferation, endothelial cell inflammation, and expansion of the plaque necrotic core, exacerbating cardiovascular damage.^202^

In summary, BCAAs, key signaling molecules and metabolic substrates, are extensively involved in various pathological processes, such as glucose metabolism disorders, through multiple mechanisms, including the modulation of mTOR signaling pathway activity, the alteration of insulin secretion, and the disruption of mitochondrial energy metabolism. Intervention strategies targeting key catabolic enzymes (e.g., BCAA aminotransferases) or related signaling pathways have emerged as promising therapeutic directions for metabolic diseases.

#### 3.3.3. Phenylacetylglutamine (PAGln)

##### 3.3.3.1. PAGln influences macrophage lipid deposition through the regulation of lipid transport and autophagy.

The gut microbiota (i.e., *Clostridium scindens* and *Gordonibacter pamelaeae*) converts dietary phenylalanine into phenylacetic acid (PAA), which enters the bloodstream and combines with glutamine in the liver/kidney to form PAGln.^203^ Chronic alcohol abuse and emotional stress can disrupt the structure of the gut microbiota and impair intestinal barrier function, leading to increased PAGln entry into the bloodstream and significantly elevated PAGln serum concentrations.^204^ PAGln was shown to upregulate the expression of receptors responsible for the uptake of oxidized low-density lipoprotein (e.g., CD36, ACAT1, and SRA) and downregulate the expression of receptors that promote cholesterol excretion, thereby increasing the total cholesterol content in macrophages and promoting lipid deposition. Furthermore, PAGln inhibits autophagy via AMPK-mTOR-ULK1 signaling, thereby increasing intracellular lipid accumulation.^205^

##### 3.3.3.2. PAGln contributes to myocardial inflammation and atrial fibrillation through the activation of the NLRP3 inflammasome.

Gut microbiota dysbiosis leads to elevated PAGln levels. PAGln activates the NLRP3 inflammasome, which promotes the release of inflammatory factors such as TNF-a, IL-1β, and IL-6. These inflammatory factors trigger an inflammatory response, disrupting the normal microenvironment of myocardial tissue and causing atrial myocyte damage and dysfunction.^206^ Moreover, the inflammatory response exacerbates atrial fibrosis, affecting atrial structure and electrophysiological properties.^207^ The release of inflammatory factors and the inflammatory response interfere with normal atrial electrophysiological activity, leading to changes such as shortened effective refractory periods and increasing the likelihood of atrial fibrillation (AF).^208^ Moreover, inflammation-mediated tissue damage and fibrosis continuously alter atrial structure, thereby providing a pathological basis for the onset and maintenance of AF.^209^

##### 3.3.3.3. PAGln modulates platelet activity and mitochondrial function to influence atherosclerosis and cellular senescence.

In human blood, isolated platelets, and carotid artery injury mouse models, PAGln can increase platelet activity and aggregation through GPCRs (including α2A, α2B, and β2 adrenergic receptors) and Ca^2+^ levels, accelerate platelet aggregation and thrombus formation, impair vascular endothelial function, and ultimately facilitate the development of atherosclerosis.^210^ Studies have reported that the structure of the gut microbiota changes with age, and the abundance of PAA-producing strains (e.g., *Ruthenibacterium lactatiformans*) increases, thus leading to a J-shaped increase in the abundance of the gut microbiota metabolite PAGln.^211^ PAGln, which is structurally similar to catecholamines, can bind to b1/b2 adrenergic receptors, activating G protein-coupled signaling, promoting cAMP generation, and activating AMPK.^212^ Activated AMPK phosphorylates the mitochondrial fission protein dynamin-related protein 1 (Drp1), promoting the recruitment of Drp1 to mitochondria and causing excessive mitochondrial fragmentation. An abnormal mitochondrial morphology further leads to a decrease in the mitochondrial membrane potential and the excessive production of ROS (e.g., superoxide anions).^213^ ROS produced by dysfunctional mitochondria directly attack nuclear DNA, inducing the formation of phosphorylated H2AX foci and activating the DNA damage response pathway.^214^ The DNA damage response activates p53 through ATM/ATR-Chk2 phosphorylation, promoting p21 expression and inhibiting cyclin-dependent kinases 4/6, ultimately arresting the cell cycle in the G1 phase.^215^^,^^216^ Moreover, persistent DNA damage induces the expression of the cyclin-dependent kinase inhibitor p16^INK4a^, inhibiting Rb phosphorylation and blocking the cell cycle through a dual mechanism, ultimately leading to irreversible cellular senescence.^217^

In summary, PAGln, a key metabolite of PAA produced by the gut microbiota, plays a significant role in the pathogenesis and progression of cardiovascular diseases such as atherosclerosis, thrombosis, and heart failure. This is primarily achieved through core mechanisms, including interference with GPCR signaling, the modulation of platelet activation, and the alteration of vascular function. Consequently, modulating the metabolic pathways of associated gut microbiota or blocking their downstream signaling pathways have emerged as novel strategies for the prevention and treatment of cardiovascular metabolic disorders.

#### 3.3.4. Hippuric acid

Hippuric acid is an amino acid metabolites. Under prolonged high-fat dietary conditions, the reduction in the abundance of the *Alistipes indistinctus* leads to a decrease in the production of its metabolite hippuric acid. This diminishes the binding of PPARγ to the ABCG2 promoter, thereby resulting in decreased uric acid excretion, exacerbated uric acid metabolic disorders.^218^

#### 3.3.5. Tyrosine

Abnormal tyrosine metabolism also contributes to lipid disorders. Intestinal gram-positive bacteria (e.g., *Lactobacillus* and *Enterococcus*) express tyrosine decarboxylase, which catalyzes the conversion of tyrosine to tyramine.^219^^,^^220^ However, certain factors, such as the consumption of a high-fat, low-fiber diet, circadian rhythm disruption, the abuse of antibiotics/nonsteroidal anti-inflammatory drugs, chronic stress, and environmental toxins, cause the attenuation of gram-positive bacteria by disrupting the microbial microenvironment, inhibiting nutrient uptake, or directly killing bacteria. The reduced abundance of gram-positive bacteria inactivates the tyramine-CaMKII-CREB/CRTC axis, leading to the upregulation of Magro gene expression in the intestine. Magro is a key regulator of lipid metabolism, and studies have shown its involvement in the hydrolysis of TGs and cholesterol esterification. The excessive digestion and absorption of lipids result in lipid overload in the liver and bloodstream, leading to dyslipidemia, which in turn disrupts lipid metabolism.^221^ This mechanism provides a theoretical basis for targeting the “microbiota-tyramine” axis in the treatment of metabolic diseases.

#### 3.3.6. Glutamate

The consumption of a high-sugar, high-fat diet or a low-fiber diet led to a reduction in *Ruminococcus* abundance, affecting the structure and function of the gut microbiota. *Ruminococcus* converts *α*-ketoisocaproate into glutamate through enzymes that breakdown fiber.^222^ Glutamate is among the primary sources of this compound in the intestine. Glutamate serves as a potent stimulator of GLP-1.^223^ A decrease in *Ruminococcus* abundance directly reduces the level of glutamate in the intestine, thereby inhibiting the synthesis and secretion of GLP-1. Insufficient GLP-1 results in the absence of “second wave activation” of insulin secretion, leading to a delayed response of pancreatic *β*-cells to glucose stimulation and further exacerbating glucose metabolism disorders.^224^

#### 3.3.7. Arginine

The synthesis of arginine in the intestine originates primarily from dietary proteins, endogenous synthesis, and protein turnover and can also be achieved through biosynthesis by the gut microbiota.^225^ Arginine can be metabolized by the gut microbiota into various metabolites, including nitric oxide (NO), gamma-aminobutyric acid (GABA), nitrite, urea, carbon dioxide, ammonia, and polyamines (such as proline, putrescine, and spermine).^225^^,^^226^ The accumulation and transformation of these metabolites in the intestine play crucial roles in regulating the acid-base balance of the intestinal environment, maintaining intestinal barrier function, and influencing immune responses. The synthesis of arginine is also influenced by various environmental factors, such as microbial community composition, pH, and oxygen concentration. Examples of arginine-synthesizing microbes include *Escherichia coli*, *Klebsiella* spp., *Pseudomonas aeruginosa*, *Pseudomonas fluorescens*, and *Bacillus subtilis*. Certain intestinal conditions, such as a low pH or an anaerobic environment, may prevent specific microorganisms from producing arginine. Recent studies have demonstrated that certain pathogenic bacteria in the gut microbiota can metabolize arginine via the arginine deiminase (ADI) pathway, which converts arginine into ornithine and releases ATP, ammonia, and CO_2_. This process generates molecules called polyamines. Polyamines act as “glue” in bacterial populations, adhering to bacterial surfaces and promoting their aggregation, ultimately leading to the formation of dense biofilm structures. These biofilm structures effectively block the entry of host immune cells and antibodies into the bacterial interior, thereby exacerbating intestinal inflammatory responses.^227^ More notably, *Helicobacter pylori* and *Salmonella enterica* weaken the host immune response by reducing the synthesis of endogenous NO by utilizing arginase. In innate immunity, arginine deficiency is often associated with abnormal expression of proinflammatory factors (e.g., the chemokines CCL2 and CXCL8), which may lead to immune cell infiltration and the exacerbation of chronic inflammation.^228^ In adaptive immunity, arginine provides an essential environment for the proliferation and activity of CD4+ T cells and CD8+ T cells. Arginine deficiency significantly impairs T-cell function, which manifests as functional attenuation and upregulated programmed death 1 (PD-1) expression, thereby compromising their ability to clear viruses and elicit effective immune responses.^229^ In mouse models, dietary supplementation with arginine significantly improved T-cell activity and enhanced antigen-specific responses to intestinal inflammation, further highlighting the importance of arginine in maintaining T-cell function.

Gut microbiota amino acid metabolic reprogramming is among the core mechanisms of host-microbe mutual adaptation and involves the regulation of microbial metabolites, signaling pathways, and the endocrine and epigenetic systems on multiple levels (Table 3). By precisely modulating microbial composition, dietary structure, and metabolic product signaling to regulate gut microbiota amino acid metabolic reprogramming, innovative prevention and treatment strategies for metabolic diseases, and inflammatory diseases can be developed.

**Table 3.** Key microbial metabolites, host receptors, and altered pathways in the context of gut microbiota amino acid metabolic reprogramming.

Microbial metabolite(s)	Full name of metabolite(s)	Related beneficial bacteria	Related harmful bacteria	Host receptor(s)	Altered signaling or metabolic pathway(s)	Terminal effect on the host
Kynurenine↑	/	Ruminococcaceae↓ Rikenellaceae↓	*Proteobacteria*↑ Enterobacteriaceae↑ *Escherichia*↑	AhR↑	AMPK-PPARα signaling pathway↓ FA *β*-oxidation↓ Lipid synthesis↑	Lipid metabolism
				AhR↑	NF-κB signaling pathway↓ T cells↓	Immunomodulation
IPA, IAA↓	3- Indolepropionic acid, indoleacetic acid	*Lactiplantibacillus* ↓ *Bifidobacterium*↓ Clostridium↓	/	AhR↓ STAT3↓	AMPK-PPARα signaling pathway↓ Lipid synthesis↑ Lipid catabolism↓ Leptin sensitivity↓	Lipid metabolism
		*Lactobacillus*↓ *Rhodococcus*↓	/	AhR ↓	GLP-1 secretion↓ Insulin secretion↓	Glucose metabolism
ICA↑	Indole-3-carboxylic acid	Bacteroidaceae↓ Lactobacillaceae↓	/	AhR↓	Nrf2-HO-1 antioxidant pathway↓	Lipid metabolism
IS↑	Indole sulfonate	/	*Escherichia coli*↑	AhR↑	TGF-β1 synthesis↑ Uric acid excretion↓	Uric acid metabolism
BCAAs↑	Branched-chain amino acids	Bacteroidetes↓	Firmicutes↑	/	mTORC1 signaling pathway↑ FA synthesis↑ TG synthesis↑ FA *β*-oxidation↓	Lipid metabolism
				IRS1↓ GLUT4↓	Insulin signaling↓ Peripheral glucose uptake↓ Insulin sensitivity↓	Glucose metabolism
				/	mTORC1 signaling pathway↑	Inflammation
PAGln↑	Phenylacetylglutamine	*Clostridium scindens*↑ *Gordonibacter pamelaeae*↑	/	CD36↑ ACAT↑ SRA↑	Lipid synthesis↑ AMPK-mTOR-ULK1 signaling pathway↑	Lipid metabolism
				/	NLRP3 inflammasome↑	Immunomodulation
Hippuric acid↓		*Alistipes indistinctus*↓		ABCG2↓	Uric acid excretion↓	Uric acid metabolism
Tyramine↓	/	*Lactobacillus*↓ *Enterococcus*↓	/	/	CREB/CRTC signaling pathway↓ Magro gene expression↑ Lipid digestion↓ Lipid absorption↑	Lipid metabolism
Glutamate↓	/	*Ruminococcus*↓	/	/	GLP-1 synthesis↓ Insulin secretion↓ Insulin sensitivity↓	Glucose metabolism
Arginine↓	/	*Escherichia coli*↑ *Klebsiella spp*.↑ *Pseudomonas aeruginosa*↑ *Pseudomonas fluorescens*↑ *Bacillus subtilis*↑ *Helicobacter pylori*↑ *Salmonella enterica* ↑	/	PD-1↑	ADI pathway↓ Endogenous NO synthesis↓	Immunomodulation

### 3.4. Gut microbiota uric acid metabolic reprogramming drives host metabolic disorders

**Figure 4.**
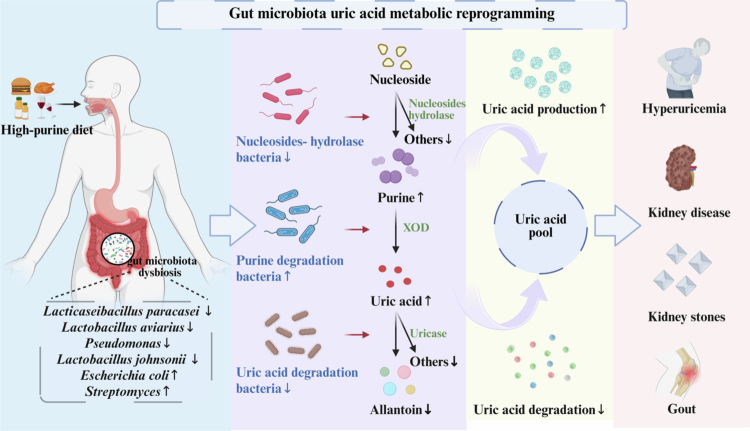
Gut microbiota uric acid metabolic reprogramming affects host uric acid production/degradation/excretion, and immune regulation, thereby driving the development of hyperuricemia and related diseases. XOD: Xanthine oxidase.

Gut microbiota uric acid metabolic reprogramming refers to the phenomenon in which the gut microbiota changes gut purine decomposition, uric acid synthesis, and decomposition, which further affect uric acid homeostasis in the host. Research on gut microbiota uric acid metabolic reprogramming provides new information for the treatment of related diseases (Figure 4).

#### 3.4.1. Uric acid

##### 3.4.1.1. Gut microbiota uric acid metabolism modulates its systemic concentrations in the host.

Uric acid, as the end product of human purine metabolism, is synthesized through a series of enzymatic reactions where nucleosides are converted into purines and subsequently transformed into uric acid.^230^^,^^231^ Gut microbiota also participated in this complex metabolic process. A high-fructose diet and excessive intake of purine-rich foods disrupt this balance, leading to dysbiosis of specific microbial communities. Probiotic bacteria (e.g., *Lactobacillus* and *Enterococcus*) are reduced, while certain opportunistic pathogens (e.g., *Escherichia coli* and *Streptomyces*) may become relatively enriched.^232^^,^^233^ This dysbiosis profoundly regulates uric acid levels by directly affecting gut uric acid metabolism.

Dysbiosis microbiota primarily affect on nucleoside hydrolase and xanthine oxidase (XOD). Nucleoside hydrolase catalyzes the breakdown of nucleosides into bases and pentose. XOD is the key enzyme in purine metabolism that generates uric acid. In a high-purine diet, the reduction of *Lactobacillus plantarum*, which produces nucleoside hydrolase RihA-C, and *Lactobacillus aviarius*, which encodes the nucleoside hydrolase ORF00069, decreases their ability to metabolize into non-absorbable substances, while degradation into purines increases, thereby increasing the host's opportunity to absorb uric acid and its precursors.^234^^,^^235^ Enriched *Escherichia coli, Proteus,* and *Streptomyces* can directly secrete XOD, promoting uric acid production.^236^^,^^237^ Additionally, facultative anaerobic bacteria in the gut microbiota can utilize oxygen molecules as final electron acceptors to generate large amounts of reactive oxygen species and secrete substantial amounts of XOD, further promoting uric acid production. In addition, dysbiosis of the gut microbiota can lead to increased intestinal permeability, resulting in chronic inflammation. Chronic inflammation is often accompanied by elevated levels of LPS and XOD activity, which may be a significant mechanism underlying hyperuricemia.^238^

In terms of uric acid degradation, most mammals oxidize uric acid into allantoin through uricase. However, humans lost uricase during long-term evolution, and the gut microbiota took over this function.^239^ Uric acid can be degraded into allantoin by the uricase secreted by gut microbiota. Additionally, gut microbiota secrete allantinase and allantinase, which further break down allantoin into allantoin acid and urea. These compounds are subsequently degraded by urease into ammonia and carbon dioxide, which are excreted from the body.^240^ Disrupted microbiota weaken the critical degradation enzyme system, thereby lead to elevated uric acid. The allantoin pathway for uric acid degradation is directly weakened by a high-purine diet, which diminishes the abundance of bacterial producers (e.g., *Lactobacillus* and *Pseudomonas*) of the requisite enzymes, uricase and allantoinase.^240^ Furthermore, a high-purine diet may compromise the alternative 2, 8-dioxopurine pathway, which can convert uric acid to SCFAs via impairment of 2, 8-dioxopurine dehydrogenase, in bacteria such as *Clostridioides difficile* and *E. coli*.^241^

In summary, gut microbiota uric acid metabolic reprogramming elevates uric acid levels through impairing gut uric acid metabolism The elevated uric acid could trigger systemic inflammation, further compromising the intestinal environment and exacerbating dysbiosis, thereby forming a self-reinforcing vicious cycle that perpetuates disease progression.

##### 3.4.1.2. Uric acid is involved in the regulation of M1 macrophage polarization, inflammatory responses, and immune activation.

Gut microbiota dysbiosis can lead to elevated host uric acid levels, which in turn directly modulates immune cell function. Specifically, uric acid significantly upregulates the expression of TNF-*α* and TLR4 in macrophages, thereby enhancing their phagocytic activity. Moreover, uric acid markedly reduces the expression levels of cluster of differentiation 206, C-X3-C motif chemokine receptor 1 (CX3CR1), and chemokine receptor 2 (CCR2). These altered expression profiles collectively drive macrophage polarization toward the proinflammatory M1 phenotype, which is accompanied by the downregulation of the urate reabsorption transporter URAT1. On the basis of these mechanisms, modulating uric acid levels can effectively alleviate immune disorder.^242^ For instance, allopurinol significantly reverses the phenotypic imbalance of macrophage polarization induced by hyperuricemia by reducing serum uric acid levels, specifically by decreasing the proportion of proinflammatory M1 macrophages while increasing the proportion of anti-inflammatory/repairing M2 macrophages, thereby ameliorating renal inflammation and injury. In summary, the pathway from gut microbiota dysbiosis to elevated uric acid levels and further to macrophage M1/M2 imbalance constitutes a clear metabolic-immune pathological axis.

##### 3.4.1.3. Uric acid influences high-altitude adaptation through the modulation of oxidative stress.

The reprogramming of gut-derived uric acid metabolism not only influences related metabolic diseases but also bolsters human adaptability to extremely hypoxic environments through orchestrated metabolic rewiring. Emerging evidence highlights the critical role of gut microbial communities in facilitating high-altitude adaptation through the regulation of gut uric acid catabolism. Under normoxic conditions, uric acid homeostasis is tightly regulated by gut microbial metabolism, but high-altitude hypoxia triggers a shift in the composition of the gut microbiota. The increased abundance of *Lachnospiraceae* promotes uric acid degradation, mitigating oxidative stress and preserving intestinal barrier function, processes that are critical for acclimatization.^243^

In summary, gut microbiota uric acid metabolic reprogramming drives the occurrence and development of hyperuricemia through various mechanisms and is closely linked with a variety of diseases (Table 4). These findings reveal the core role of the gut microbiota in uric acid metabolic balance, providing new ideas for the treatment of hyperuricemia and related diseases.

**Table 4.** Key microbial metabolites, host receptors, and altered pathways in the context of gut microbiota uric acid metabolic reprogramming.

Microbial metabolites	Related beneficial bacteria	Related harmful bacteria	Host receptors	Related enzyme	Altered signaling or metabolic pathway	Host terminal effect
Uric Acid↑	*Lactobacillus plantarum*↓ *Lactobacillus aviarius*↓	/	/	Nucleoside hydrolase↓	Nucleoside hydrolysis pathway↓	Uric acid metabolism
	*Lactobacillus johnsonii*↓	*Escherichia coli*↑ *Proteus*↑ *Streptomyces*↑	/	Xanthine oxidase↑	Purine degradation pathway↑	
	*Lactobacillus*↓ *Pseudomonas*↓	*/*	/	Uricase↓	Uric acid degradation pathway↓	
	*Lactobacillus*↓	*/*	CX3CR1↓ CCR2↓ URAT1↓		Pro-inflammatory M1 phenotype	Immunomodulation
	Lachnospiraceae↓				Uric acid degradation pathway↓	Oxidative stress

## 4. Discussion

Metabolic diseases are a group of chronic disorders caused by host metabolic reprogramming. These conditions have become major global health challenges.^1^ The gut microbiota, often called the “second genome”, continuously interacts with the host’s metabolism and immune system through its metabolites. Imbalances in the gut microbiota are key drivers of metabolic disorders. An imbalance in the gut microbiota leads to significant reprogramming of gut metabolic pathways, including lipid, amino acid, uric acid, and glucose metabolism.^43^^,^^244-246^ Such reprogramming systematically disrupts systemic metabolic and immune homeostasis through multiorgan communication chains such as the gut-liver, gut-brain, and gut-immune axs, ultimately accelerating the onset and progression of metabolic diseases.^247-250^ Therefore, studying gut microbial metabolic reprogramming provides a novel framework for understanding and treating metabolic diseases.

This review systematically describes the central role of gut microbiota metabolic reprogramming in the pathogenesis of metabolic diseases. By analyzing the reprogramming of gut microbiota-related lipid, amino acid, uric acid, and glucose metabolism, this study revealed that dynamic changes in the gut microbiota metabolic system are not isolated events but rather complex processes involving coordinated disorder of multiple metabolic and immune pathways, which collectively drive the development and progression of metabolic diseases in the host.^251^ The mechanisms by which gut microbiota metabolic reprogramming induces metabolic diseases exhibit a dual effect. On the one hand, it manifests as reduced absorption of beneficial microbiota-derived metabolites; on the other hand, it is characterized by the production of new harmful substances due to abnormal microbiota-mediated metabolism.^252^^,^^253^ Insufficient absorption of beneficial metabolites can directly weaken host metabolism and immune regulation. For instance, SCFAs such as propionate and butyrate are insufficiently absorbed because of reduced bacterial production, resulting in weakened inhibition of liver cholesterol synthesis and decreased activation of AMPK signaling, which leads to altered cholesterol metabolism and inhibited lipid catabolism, promoting the accumulation of lipids in the liver and peripheral tissues.^254^ Intestinal and systemic immune homeostasis are also affected by SCFAs. SCFAs maintain an anti-inflammatory environment by promoting the differentiation of regulatory T cells and inhibiting the expansion of Th17 cells. SCFAs deficiency disrupts the Treg/Th17 balance, driving inflammatory progression. Such defects in the absorption of critical metabolites, analogous to “signal loss” in the metabolic regulatory network, directly disrupt the balance between energy metabolism, lipid synthesis, and lipid decomposition.^254^ On the other hand, it is manifested primarily in metabolic-immune cascade reactions triggered by harmful substances derived from abnormal microbiota.^253^ When the microbiota structure is imbalanced, the overgrowth of proinflammatory bacteria promotes the massive production of toxic metabolites such as LPS, which activate the TLR4/NFκB pathway to drive macrophages to polarize toward the proinflammatory M1 phenotype and induce systemic low-grade inflammation.^255^ Furthermore, TMAO, a metabolite produced by the gut microbiota, not only promotes macrophage M1 polarization and foam cell formation but also activates the NLRP3 inflammasome, thereby amplifying inflammatory signals, exacerbating insulin resistance, and impairing vascular endothelial function.^82^^,^^83^ Notably, insufficient absorption of beneficial metabolites weakens the protective function of the intestinal barrier, creating conditions conducive for the production and diffusion of harmful substances.^254^ Conversely, inflammation and metabolic stress induced by harmful substances further inhibit the proliferation and metabolic activity of beneficial bacteria, exacerbating disruptions in the synthesis and absorption of beneficial metabolites.^253^ This synergistic disorder of metabolic and immune pathways makes gut microbiota metabolic reprogramming a core driver of the progression of metabolic diseases. In summary, studying metabolic reprogramming in the gut microbiota contributes to our understanding of the “metabolism-immunity” axis centered on the gut microbiota and highlights that targeting this reprogramming process holds significant potential for early intervention and novel therapeutic development.

### 4.1. Therapeutic (and diagnostic) implications

The gut microbiota metabolic reprogramming represents a promising target for the early intervention and treatment of metabolic diseases. First, it holds therapeutic potential. Multiple studies have demonstrated that supplementation with *A. muciniphila* modulates the BA-microbiota axis, significantly reducing body weight, fat mass, and metabolic endotoxins while improving insulin sensitivity.^256^ Oral administration of *Lacticaseibacillus casei LC2W* notably improved weight control, glucose and lipid metabolism, the levels of inflammatory and oxidative stress markers, and SCFA production, with positive effects observed in vivo in terms of colitis prevention and the alleviation of hypertension.^257^ The synergistic actions of multiple strains, including *Limosilactobacillus reuteri NCIMB 30242*, *Lactiplantibacillus plantarum UALp05*, and *Bifidobacterium animalis* subsp. *lactis B420,* can improve blood lipid levels, blood glucose levels, and blood pressure within a short period.^258^ Supplementation with lactate-producing probiotics increases intestinal lactate levels, improves hepatic gluconeogenesis regulation, and reduces free FA accumulation caused by excessive fat breakdown.^259^ Another study indicated that *Lactobacillus murus* and its metabolite lactate are crucial for the hyperproliferation of the colonic epithelium following prolonged fasting and subsequent refeeding, suggesting that lactate is also utilized by epithelial cells.^260^ Such interventions can regulate metabolic pathways at the source by restoring microbial metabolic balance. Second, metabolic reprogramming has low toxicity and does not directly affect other organs.^261^ As key participants in metabolic reprogramming, gut microbiota-derived metabolites such as SCFAs and BAs exert therapeutic effects by regulating host metabolic signaling pathways.^262^ This physiology-based intervention approach is more similar to the body’s metabolism than traditional drugs are, thereby significantly reducing the risk of toxicity.^261^ Thus, gut microbiota metabolic reprogramming can serve as a target for early disease prevention and treatment.

Metabolites and bacteria related to gut microbiota metabolic reprogramming can serve as diagnostic markers for diseases.^263^ Patients with metabolic diseases exhibit characteristic changes in gut microbiota metabolic reprogramming.^264^ For instance, individuals with obesity or MAFLD often have reduced levels of SCFA-producing bacteria, elevated levels of LPS, and abnormal levels of BCAAs, with these changes typically preceding the onset of clinical symptoms.^265^ Detecting the gut microbiota composition or levels of specific metabolites (e.g., SCFAs, lactate, and uric acid) enables early disease identification and risk assessment.^192^

In conclusion, gut microbiota metabolic reprogramming serves as a pivotal link between microbial ecology and host metabolic homeostasis and plays a driving role in the pathogenesis and progression of metabolic diseases. This mechanism not only provides a novel perspective for understanding metabolic disorders but also opens new avenues for early diagnosis and precision intervention by targeting microbial structure modulation and restoring metabolite balance and holds promise for achieving root-level regulation of metabolic homeostasis.

### 4.2. Challenges

Gut microbiota metabolic reprogramming offers broad prospects for reinterpreting metabolic diseases, yet translating these findings into clinical practice remains challenging because of a series of interconnected core issues. These challenges involve primarily high interindividual variability, the lack of systematic multipathway mechanistic studies, and methodological limitations in establishing causality and quantifying microbial metabolic effects. Overcoming these obstacles is crucial for advancing the field toward effective, precision personalized therapies.

Interindividual variability represents a critical limitation in elucidating regulatory mechanisms and clinical efficacy. The composition of the gut microbiota significantly differs across individuals, populations, and dietary patterns, stemming from host genetic background, long-term dietary habits, drug exposure, and functional variations at the microbial strain level, collectively contributing to the heterogeneity of host-microbiota interactions.^266^^,^^267^ Host genetic polymorphisms (e.g., variants in FXR and nucleotide-binding oligomerization domain-containing protein 2 (NOD2) genes) can alter responses to microbial signals and microbial community structure.^268^^,^^269^ Diet serves as the primary driver shaping microbial communities, with high-fat, low-fiber Western diets contrasting markedly with traditional high-fiber diets in terms of microbial community structure.^270^ Drugs (e.g., metformin and tetracycline) can directly or indirectly influence host metabolism through the microbiota.^271^^,^^272^ Different strains of the same species may also exhibit distinct functional profiles. For instance, only certain *E. coli* strains produce carcinogens, while others lack pathogenicity.^273^ These factors collectively account for the variability in the effects of the same intervention across individuals. Therefore, addressing the high degree of interindividual variability, rooted in genetics, diet, and microbial strain specificity, is a fundamental prerequisite for translating gut microbiota metabolic reprogramming into predictable and effective therapies.

Mechanistic research on gut microbiota metabolic reprogramming is limited by the absence of a systematic perspective. Most studies to date have concentrated on isolated metabolic pathways or a handful of “star” microbial metabolites, such as SCFAs and BAs. This fragmented approach overlooks the multilevel, multipathway regulatory networks that function in vivo, in which microbial metabolites concurrently modulate host energy metabolism, immune responses, and neuroendocrine signaling through dynamic and interconnected crosstalk. Such a reductionist framework not only risks oversimplification of the underlying mechanisms but also may lead to an overestimation of the therapeutic potential of microbiota-targeted interventions. In summary, advancing this field requires a shift from isolated pathway analyzes toward integrative systems-level approaches that can capture the complex, network-based interactions between the gut microbiota and host metabolism. Only through such a holistic perspective can we accurately elucidate the underlying mechanisms and design effective, context-aware therapeutic strategies.

Furthermore, methodological and technical constraints further impede progress in gut microbiota metabolic reprogramming research. Chief among these is the field’s continued heavy reliance on correlative studies, which complicate the establishment of clear causal mechanisms linking specific microbial functions to host metabolic outcomes. Translational relevance is additionally limited by species differences between animal models and humans. Differences in physiology, immune function, and native microbiota composition reduce the direct applicability of preclinical findings to human populations. At the analytical level, metabolic quantification techniques, such as those used to detect SCFAs, face ongoing challenges in terms of sensitivity, reproducibility, and standardization, hindering accurate cross-study comparisons. Collectively, these methodological gaps underscore the necessity of developing more causally informative models, improving translational fidelity, and refining analytical and interventional tools to advance the field toward robust, clinically applicable insights.

In the process of advancing toward clinical application, in addition to the aforementioned challenges, the interventions themselves also face uncertainty in terms of translational parameters and efficacy bottlenecks. Although interventions such as probiotic or dietary fiber administration or microbiota transplantation have shown moderate positive effects on metabolic parameters such as the levels of blood glucose and blood lipids, their efficacy, stability and intensity often fall short of those of traditional targeted drugs. Furthermore, key translational parameters, including the optimal dosage, treatment duration, long-term safety, and potential off-target effects, remain poorly defined, introducing uncertainty into clinical protocol design.

### 4.3. Future perspectives

On the basis of the identified challenges and research gaps, the field of gut microbiota metabolic reprogramming research stands at the precipice of a pivotal shift from describing associations to enabling prediction and personalized intervention. To realize this future and harness the therapeutic potential of targeting the microbiome for metabolic diseases, research must evolve along several key, forward-looking dimensions. The following four interconnected directions outline a strategic path to transform our understanding of gut microbiota metabolic reprogramming in hosts into a predictive and actionable science, guiding the development of next-generation diagnostics and tailored therapies.

To achieve personalized gut microbiota metabolic reprogramming-based interventions for metabolic diseases, future research should focus on building a data-driven predictive framework. Central to this effort is the integration of multidimensional data, such as from metagenomics and targeted metabolomics, to systematically decipher gut microbiota metabolic reprogramming-host interaction networks. By performing simultaneous profiling of fecal metagenomes and serum/fecal targeted metabolites in large-scale cohorts, an individual-specific “gut microbiota metabolic reprogramming-host metabolic map” can be constructed. Using machine learning algorithms, this map will enable the definition of biologically distinct “host-microbiota metabolic subtypes” and the development of “personalized gut microbiota metabolic reprogramming signatures”. The key application of such a model lies in its predictive capacity, which can prospectively determine how a given individual will respond to a specific dietary regimen (e.g., a high-fiber or low-carbohydrate diet) or to various probiotic formulations. This strategy will advance clinical practice from one-size-fits-all recommendations toward “precision nutrition” and “precision microbiota modulation,” ultimately transforming gut microbiota metabolic reprogramming research from a descriptive field into a predictive and actionable science that guides individualized therapy.

Then, a translational research pipeline spanning from mechanism to strain-level application should be established. This will require combining basic mechanistic studies of gut microbiota metabolic reprogramming with functional validation at the strain level and the rational design of engineered microbial strains. When integrated with host genotyping, this pipeline will enable the development of personalized microbial community modulation protocols that account for both microbial function and host genetic background, thereby addressing the critical issue of interindividual variability in treatment response.

Large-scale longitudinal clinical cohort studies are essential to move beyond snapshots of association. Such studies should track the dynamic progression of gut microbiota metabolic reprogramming across the stages of metabolic disease. Through repeated multiomics profiling, researchers can map evolving molecular regulatory networks, clarify the upstream-downstream relationships between key metabolites (e.g., SCFAs, BAs, and amino acid derivatives), and elucidate how different signaling pathways interact synergistically or antagonistically during disease development and intervention.

Finally, efforts to identify clinically actionable microbial targets should intensify. Beyond mechanistic studies, research should focus on discovering specific microbial taxa, genes, or metabolic pathways that hold diagnostic, prognostic, or therapeutic value. Validating these targets in independent cohorts and linking them to clear clinical endpoints will be crucial for developing reliable biomarkers and effective microbiota-based interventions that can be integrated into standard clinical practice for metabolic disease management.

## 5. Conclusions

This review systematically elucidates that gut microbiota metabolic reprogramming serves as a crucial bridge linking dysbiosis to metabolic diseases. It exhibits significant systemic characteristics, with complex interactive networks formed among different metabolic pathways. These pathways collectively drive disease progression through a tripartite regulatory “gut microbiota-metabolite-host” model. This research provides a novel perspective for understanding the pathogenesis of metabolic diseases and elucidating the molecular mechanisms through which the gut microbiota influences host metabolic homeostasis through the regulation of lipid, glucose, amino acid, and uric acid metabolic networks. These research findings will facilitate the development of novel preventive and therapeutic approaches for metabolic diseases from the “microbiome-metabolome” perspective, providing theoretical foundations and practical guidance for the realization of personalized medicine. Despite existing challenges such as model translation and interindividual variability in current research, as a result of technological advancements and deeper mechanistic insights, therapeutic strategies targeting gut microbiota metabolic reprogramming hold promise as a significant breakthrough in the management of metabolic diseases.

## Data Availability

Data sharing is not applicable to this article, as no new data were created or analyzed in this study.

## Abbreviations

T2D: Type 2 diabetes
MAFLD: Metabolic dysfunction-associated fatty liver disease
FA: Fatty acid
c-Myc: MYC proto-oncogene
SCFAs: Short-chain fatty acids
BAs: Bile acids
5-HT: 5-Hydroxytryptamine
TMAO: Trimethylamine *n*-oxide
BCAAs: Branched-chain amino acids
LPS: Lipopolysaccharide
FXR: Farnesol X receptor
DCA: Deoxycholic acid
CYP7A1: Cholesterol 7α-hydroxylase
SREBP-1c: Sterol regulatory element-binding protein-1c
SCD1: Stearoyl-CoA desaturase 1
FAS: Fatty acid synthase
ACC: Acetyl-CoA carboxylase
PPARα: Peroxisome proliferator-activated receptor α
CPT1: Carnitine palmitoyltransferase1
CD36: Cluster of differentiation 36
TG: Triglyceride
LAC: Lithocholic acid
GPR: G protein-coupled receptor
GLP-1: Glucagon-like peptide-1
TGR5: G protein-coupled bile acid receptor 1
GLUT: Glucose transporter
TNF-α: Tumor necrosis factor-alpha
IL-1β: Interleukin-1beta
TLR2: Toll like receptor 2
NF-κB: Nuclear factor kappa-B
VDR: Vitamin D receptor
TMA: Trimethylamine
FMOs: Flavin monooxygenases
CKD: Chronic kidney disease
HMGCS1: Hydroxymethylglutaryl-CoA synthase 1
ABCG: Adenosine triphosphate-binding transporter G superfamily member
NPC1L1: Niemann-pick c1-like protein 1
IRS: Insulin receptor substrate
PEPCK: Phosphoenolpyruvate carboxykinase
G6PC: Glucose-6-phosphatase
AAAs: Aromatic amino acids
NLRP3: NOD-like receptor thermal protein domain associated protein 3
LCFAs: Long-chain fatty acids
2-OG: 2-Octagenoate
ChREBP: Carbohydrate response element binding protein
SPT: Serine palmitoyltransferase
PP2A: Protein phosphatase 2 A
HDACs: Histone deacetylases
UCP1: Uncoupling protein 1
URAT1: Urate transporter 1
SUCNR1: Succinate receptor 1
HIF-1α: Hypoxic inducible factor-1α
RNS: Reactive nitrogen species
IBD: Inflammatory bowel disease
CYP2E1: Cytochrome P450 2E1
IAA: Indole acetic acid
ILA: Indolelactic acid
IPA: 3-Indolepropionic acid
AhR: Aryl hydrocarbon receptor
IDO1: Indoleamine 2, 3-dioxygenase
MHS: Metabolic-associated hepatic steatosis
LBP: Lipopolysaccharide-binding protein
STAT3: Signal transducer and activator of transcription 3
ICA: Indole-3-carboxylic acid
Nrf2: Nuclear factor erythroid 2-related factor 2
IS: Indole sulfonate
S6K1: S6 kinase 1
PAGln: Phenylacetylglutamine
PAA: Phenylacetic acid
AF: Atrial fibrillation
Drp1: Dynamin-related protein 1
NO: Nitric oxide
GABA: Gamma-aminobutyric acid
ADI: Arginine deiminase
PD-1: Programmed death 1
XOD: Xanthine oxidase
CX3CR1: C-X3-C motif chemokine receptor 1
CCR2: Chemokine receptor 2
*F. intestini*: *Fusimonas intestini*
*A. muciniphila*: *Akkermansia muciniphila*
